# Upcycling Wood Waste into Solar-Driven Regenerative Sorbent for Direct Air Capture

**DOI:** 10.1021/acssuschemeng.5c12124

**Published:** 2026-03-09

**Authors:** Man Qi, Bo Pang, Aji P. Mathew, Zoltán Bacsik, Niklas Hedin, Jiayin Yuan

**Affiliations:** a Department of Chemistry, 7675 Stockholm University, Stockholm 10691, Sweden; b Department of Food Science and Technology, 37580 National University of Singapore, Science Drive 2, Singapore 117542, Singapore; c Institute of Chemistry, University of Miskolc, 3515 Miskolc, Hungary

**Keywords:** Direct air capture, Lignocellulosic biomass utilization, Amine-functionalized adsorbents, Photothermal regeneration, Deep eutectic solvent

## Abstract

Direct air capture (DAC) is a promising negative-emission technology for mitigating climate change caused by excessive atmospheric CO_2_ emissions. Amine-functionalized solid adsorbents exhibit a strong affinity for CO_2_ in ambient air, making them attractive for DAC systems. However, their regeneration for reuse typically requires a high energy use during thermal swing processes. Herein, we upcycle wood waste into a DAC adsorbent that can release CO_2_ via solar light irradiation for an energy-saving DAC process. Importantly, the as-synthesized adsorbent *in situ* preserves lignin, enabling photothermal heating without addition of photothermal fillers into a complex composite. The as-synthesized adsorbent exhibits a CO_2_ uptake of 1.84 mmol/g at 25 °C, rapidly reaching 50% of its capacity within 7 min, and releasing 50% of CO_2_ in 22 min at 67 °C under solar illumination. Moreover, this study found the presence of water vapor enhances the CO_2_ adsorption capacity of the adsorbent, making it particularly advantageous for CO_2_ capture under humid air conditions. This work demonstrates a straightforward approach for developing solar-driven regenerative CO_2_ adsorbents based to a large fraction on waste lignocellulosic biomass, offering a promising pathway to sustainable and energy-efficient DAC.

## Graphical abstract

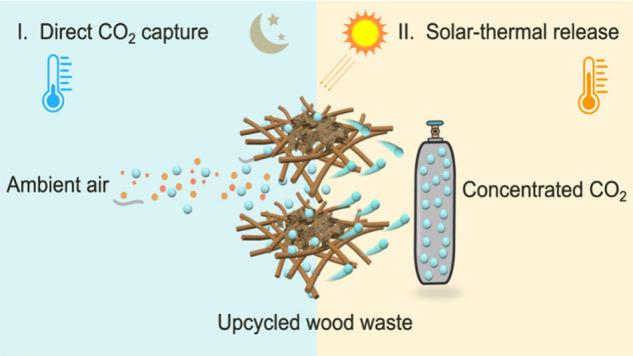

## Introduction

Direct air capture (DAC) is a promising negative-emission technology that favors direct removal of carbon dioxide (CO_2_) from the atmosphere. It can be deployed independently of CO_2_ emission source, particularly in locations with abundant and low-cost renewable energy.^1–4^ DAC systems typically operate via a reversible adsorption–desorption cycle, where sorbents selectively capture low-concentration CO_2_ in ambient air and are regenerated for reuse by applying heat (temperature swing adsorption, TSA), reducing pressure (pressure swing adsorption, PSA), altering humidity or pH.^5,6^ Amine-functionalized solid sorbents are among the most widely used adsorbents in DAC applications because of their high affinity for CO_2_ under ultradilute conditions, high amine density, and suitable heat of adsorption.^7–9^ However, solid amine sorbents also face significant challenges, especially in terms of high energy usage, thus high cost during sorbent regeneration.^10^ Because CO_2_ is chemically bonded to the amine groups, desorption typically requires external heating to release the captured CO_2_.^11,12^

To reduce the fossil fuel-based energy usage for regeneration, recent studies have explored the introduction of photothermal fillers to assist CO_2_ desorption using renewable solar energy.^13^ This strategy involves incorporation of photothermal materials, such as polydopamine,^14^ carbon black,^15^ carbon nanotubes,^16^ graphene oxide,^17^ MXene,^18^ metallic nanocrystals,^19^ and so on into the sorbent matrix. These materials can efficiently absorb sunlight and convert it into localized heat, enabling light-triggered CO_2_ desorption at a lower energy cost. Despite this promising prospective, such approaches suffer from certain limitations; for example, the addition of photothermal fillers derived from petrochemical sources may negatively affect the CO_2_ adsorption performance of the sorbent. The fabrication complexity and cost constrain the scalability and practical deployment of the sorbent. Considering that annual global CO_2_ emissions are on the gigaton scale, developing renewable and cost-effective photothermal sorbents is essential.

Lignin, as the most abundant natural aromatic biopolymers on Earth, has a molecular structure that enables strong π–π stacking. This interaction not only facilitates nonradiative energy migration but also promotes efficient photothermal conversion.^20^ These characteristics endow lignin with good thermal stability, and efficient and selective absorption of UV light.^21^ Wood is an abundant and renewable natural biomass resource, primarily composed of cellulose (40–50%), hemicellulose (10–30%), and lignin (20–30%) in mass.^22,23^ Lignin is typically removed as a waste residue during wood fractionation processes to isolate pure cellulose for materials applications, which underutilizes lignin in the design of functional materials.^24–26^ Surprisingly, lignin has not yet been reported as a photothermal component in solid adsorbents for DAC applications.

Herein, a sustainable DAC adsorbent with efficient solar-thermal regeneration capability was prepared from wood waste, where the retained cellulose provides a functionalizable framework and lignin acts as an effective photothermal component. The as-synthesized adsorbent exhibited a CO_2_ uptake of 1.84 mmol/g at 25 °C, with fast adsorption and desorption kinetics. Under simulated air, it exhibited high selectivity for CO_2_ over N_2_ and O_2_, and the CO_2_ uptake capacity across different humidity levels was also investigated. When tested with outdoor air in Stockholm, Sweden, the adsorbent achieved 10 consecutive CO_2_ uptake cycles with 0.2 mmol/g at 25 °C and desorbed CO_2_ completely at 67 °C under light irradiation, being 20–30 °C lower than conventional thermal swing processes.

## Experimental Section

### Materials and Chemicals

Birch wood powder (60–120 mesh) was used as the starting material. Choline chloride (C_5_H_14_ClNO, ≥98%), oxalic acid dihydrate (H_2_C_2_O_4_·2H_2_O, ≥99%) and *tert*-butanol (*t*BuOH, ≥99%) were acquired from Sigma-Aldrich. 3-Aminopropyl­(diethoxy)­methylsilane (APDMS, 97%) was purchased from Thermo Scientific. All chemicals were used without further purification.

### Preparation of DES

Deep eutectic solvent (DES) was prepared following the method reported by Xia et al.^27^ Choline chloride and oxalic acid (1:1 molar ratio) were mixed at 80 °C until a homogeneous and transparent liquid formed. The DES mixture was then cooled to room temperature for subsequent use.

### Preparation of Lignocellulosic Slurry and Lignocellulosic Powder

Wood powder (WP) and DES (at a 1:15 mass ratio) were mixed and heated at 110 °C for 2 h. The resulting brown liquid was diluted with distilled water (1:10 volume ratio) and then homogenized (T18 digital ULTRA-TURRAX, IKA) for 10 min. The mixture was stirred at room temperature for another 2 h. The cellulose–lignin mixture was repeatedly washed with excess distilled water and centrifuged to remove residual DES, yielding a lignocellulosic slurry with approximately 15 wt % solid content for further amine-functionalization. The lignocellulosic powder (denoted as WP-D) was obtained by freeze-drying the slurry. The chemical composition of WP and WP-D was shown in Table S1.

### Preparation of Amine-Functionalized Adsorbent

The lignocellulosic slurry was dispersed in *tert*-butanol at a mass ratio of 1:4 and heated to 90 °C under stirring. Next, APDMS was added at a mass ratio of 1:2 to the slurry. The mixture was stirred at 110 °C for 24 h, and then it was washed by tert-butanol to remove residual APDMS. The mixture was then freeze-dried, resulting in amine-functionalized adsorbent (denoted as WP-D-NH_2_). Figure S1 shows the photographs of WP, WP-D, and WP-D-NH_2_.

### Characterization

Powder X-ray diffraction (XRD) patterns of the samples were collected using a Bruker D8 Advance diffractometer (USA) with Cu Kα radiation (λ = 1.5418 Å, 40 kV, 40 mA), scanned over a 2θ range of 5° to 60°. Infrared (IR) spectra of the samples were recorded on a Varian 670-IR spectrometer (Varian, USA) equipped with a diamond attenuated total reflectance (ATR) element and a room-temperature detector. For characterization of the as-synthesized materials, IR spectra for WP, WP-D, and WP-D-NH_2_ were collected after synthesis without any pretreatment (spectra shown in the main text). In order to detect the formed species with CO_2_, IR spectra of WP-D-NH_2_ before and after CO_2_ uptake/release were collected under three conditions: (i) the as-synthesized WP-D-NH_2_ before CO_2_ uptake measured without pretreatment, (ii) the CO_2_-saturated WP-D-NH_2_ obtained after the CO_2_ gravimetric adsorption, and (iii) the CO_2_-desorbed WP-D-NH_2_ collected after thermal desorption to remove CO_2_ (spectra shown in the Supporting Information). All measurements were performed *ex situ*. The C, H, N and O elemental compositions of samples were determined by Elementar, UNICUBE, Germany. The morphological features of the samples were studied by field-emission scanning electron microscopy (FESEM) (JEOL JSM-IT800, Tokyo, Japan). Thermogravimetric analysis (TGA) of the samples was performed using a Discovery TG instrument under a dry nitrogen atmosphere. The weight loss of samples was recorded as the temperature increased from 25 to 800 °C at a heating rate of 10 °C/min. The nitrogen (N_2_) adsorption–desorption isotherms of the samples at −196 °C were recorded on a Micromeritics ASAP 2020 (accelerated surface area and porosimetry system, Norcross, USA). All samples were degassed at 80 °C for 8 h under dynamic vacuum (<0.01 Torr) before measurement. The specific surface areas of the samples were determined by the Brunauer–Emmett–Teller (BET) equation.

### Analysis of Optical and Photothermal Properties

The transmittance and reflectance spectra of the samples were assessed using a UV–vis spectrophotometer (Agilent Cary 5000 UV–vis–NIR instrument) across the wavelength range of 200–800 nm. An integrated sphere equipped with a specialized fluorine-based polymer (PTFE) served as a reference to collect the reflected light. Light absorption was calculated by subtracting the combined transmission and reflection from the incident light intensity.^28,29^ Specifically, absorption (A%) = 100 – transmittance (T%) – reflectance (R%). Simulated solar irradiation was provided by a MiniSol LED solar simulator (Newport), and the simulated sunlight was directed perpendicularly to the sample surfaces. Solar irradiance was measured using a data-logging solar power meter (ISM 410, RS Components Ltd.). The surface temperature of the samples was monitored by using a thermal infrared camera (Testo 890). Note that all samples were pressed into pellets (Figure S2) for the measurement of transmittance and reflectance spectra and solar heating measurement, and each test was repeated 3 times.

### Gas Sorption Measurement

The CO_2_, N_2_, O_2_, and Ar volumetric sorption capacities of the samples at the desired temperature were determined by Micromeritics TriStar II Plus under pressure from 0 to 1 bar. H_2_O vapor sorption measurement at 25 °C was conducted on Micromeritics 3Flex under pressure from 0 to 0.03 bar (H_2_O saturation pressure at 25 °C). All samples were degassed at 80 °C for 8 h under dynamic vacuum (<0.05 Torr) before measurement.

### Gas Sorption Kinetics

The simultaneous thermal analyzer (STA, 449 F5 Jupiter, Netzsch) was used to monitor the CO_2_ gravimetric adsorption of adsorbent WP-D-NH_2_, and the CO_2_ adsorption–desorption kinetics under controlled temperature profiles. Prior to the experiments, the furnace temperature and calorimeter heat flow were calibrated. In a typical adsorption–desorption experiment, the sample was predried at 80 °C for 90 min under N_2_, then cooled to 25 °C before introducing CO_2_ for adsorption. After adsorption, the CO_2_-saturated adsorbent was heated to the desired temperature (e.g., 80 and 100 °C) under N_2_ to achieve desorption. Additionally, the desorption behavior of the sample was observed through differential scanning calorimetry (DSC) using a DSC 3+ thermal analysis system (Mettler Toledo). Approximately 5 mg of a CO_2_-saturated sample (from STA) was heated from 30 to 120 °C under a constant nitrogen flow (100 mL/min) at a heating rate of 1 °C/min, and the heat flow was recorded.

### Moisture Uptake

The moisture uptake of the WP-D-NH_2_ at 25 °C and different relative humidity (20, 40, 60, and 80 RH%) were recorded using an analytical balance (BCE224-1S, Sartorius, Germany) placed inside a temperature- and humidity-controlled chamber (EVO, Climacell, USA). The balance was connected to a computer that recorded mass changes every 5 min for a total duration of 6 h. Prior to measurement, the sample was dried in an oven (Binder, Germany) at 80 °C overnight.

### Breakthrough Experiments

The CO_2_ uptake performance from simulated air was studied through a multicomponent adsorption breakthrough curve analyzer (BSD-MAB, Bei Shi De Instrument, Beijing, China). A schematic of the experimental setup is provided in Figure S3. Simulated dry air was prepared by mixing 400 ppm of CO_2_ with a O_2_/N_2_ mixture in a 1:4 volume ratio. Simulated wet air was obtained by introducing water vapor into the simulated dry air to get the desired different relative humidity. In a typical breakthrough experiment, the sample was packed into a quartz fixed-bed reactor with an inner diameter of 4 mm and a length of 42 mm. Prior to the breakthrough experiment, the sorbent was purged under a 50 sccm flow of He gas at 100 °C for 2 h. Subsequently, the simulated air with a total flow rate of 25 sccm was introduced from the inlet, passing through the sorbent where adsorption occurred at the selected temperatures and exiting through the outlet. Breakthrough curves were obtained by monitoring of the outlet concentration of CO_2_, N_2_, O_2_, and H_2_O using a mass spectrometer gas analyzer. The equilibrium dynamic adsorption capacity of the adsorbent was determined from the resulting breakthrough profiles.

## Results and Discussion

In this study, deep eutectic solvents (DESs) comprising choline chloride and oxalic acid were used to deconstruct waste wood (Figure 1). DESs are eutectic mixtures composed of certain hydrogen bond acceptors and hydrogen bond donors^30^ and are considered as environmentally friendly solvents due to their low toxicity, biodegradability.^31,32^ They are also easy to prepare and have tunable physicochemical properties. Hemicellulose is unstable in acidic conditions with elevated temperature, acidic DES can effectively break linkages in the lignin-carbohydrate complexes, promoting the dissolution of hemicellulose and lignin.^33–35^ The role of DES in this study is disrupting the crystalline network of cellulose while simultaneously dissolve lignin and hemicellulose in wood waste.^36,37^ The hydrophobic lignin, initially dissolved in the DES, was regenerated *in situ* as a precipitate upon the addition of polar H_2_O.^27^ After washing with H_2_O repeatedly to remove residual DES, the lignin was retained on the cellulose scaffold, forming a cellulose-lignin slurry. Subsequently, silane-based amination was then used to graft and polymerize the aminated organosilane monomer onto hydroxyl-rich cellulose, aiming to enhance CO_2_ adsorption capacity. This process involves the hydrolysis of aminosilanes to generate silanol groups, followed by polymerization of the monomers and condensation on hydroxyl groups on the cellulose surface to form Si–O–C linkages.^38,39^

**Figure 1:**
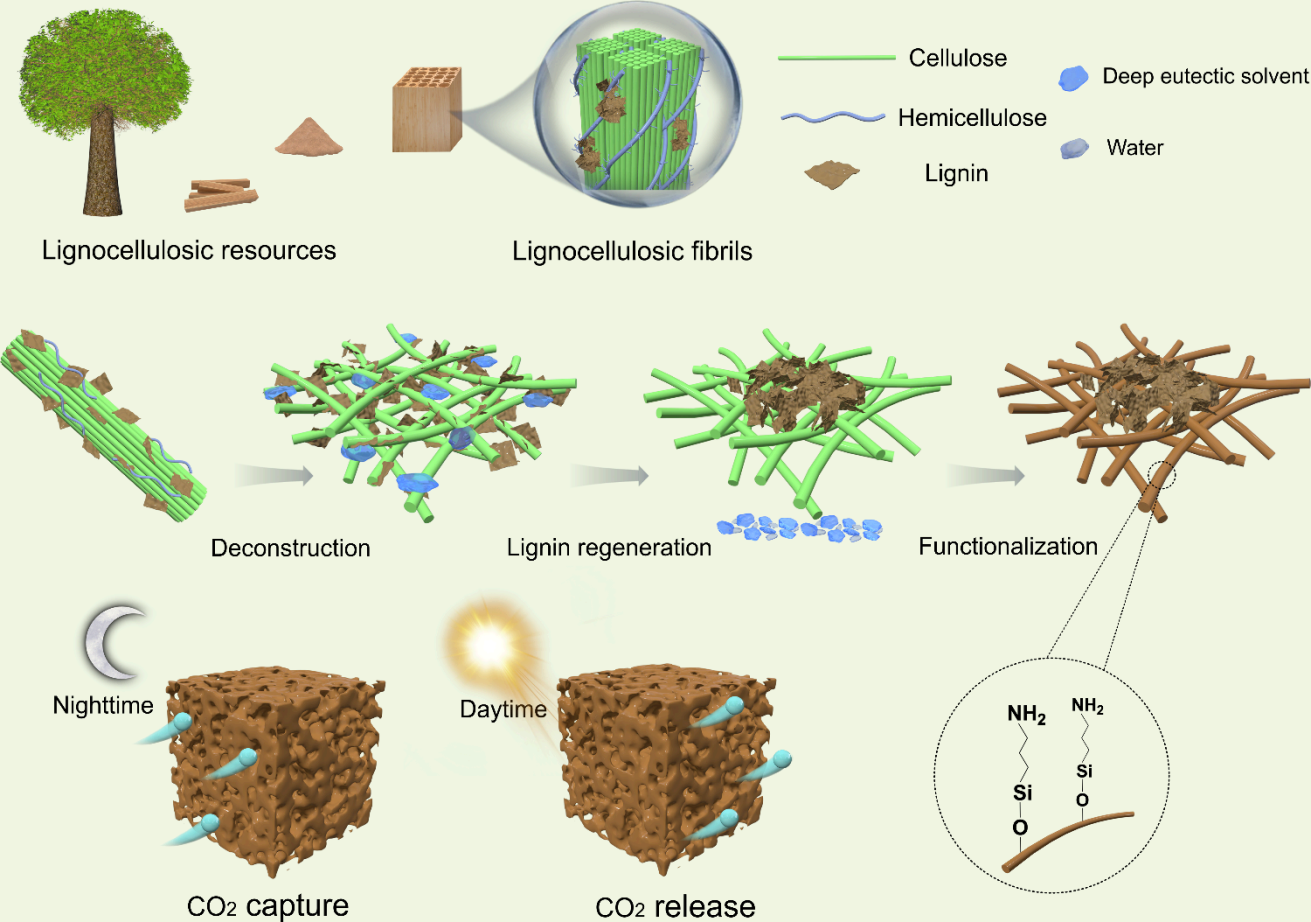
Scheme of the preparation procedure of the photothermal CO_2_ adsorbent from wood waste.

XRD was used to investigate phase changes in the wood powder (WP), lignocellulosic powder (WP-D), and amine-functionalized adsorbent (WP-D-NH_2_). All samples exhibited similar diffraction peaks (2θ = 16.5°, 22.4°, and 34.7°) indicative of the (110), (200), and (004) crystallographic planes, respectively (Figure 2a).^40,41^ WP had sharp and intense peaks, indicating a high degree of crystallinity, while the peaks of the WP-D became broader and less intense, suggesting partial disruption of the crystalline cellulose domains by breaking the native hydrogen-bond network. Further amination led to an additional decrease in the crystallinity of WP-D-NH_2_, because the hydroxyl groups on the cellulose reacted with the aminated organosilane monomer. IR spectroscopy was used to characterize the functional groups (Figure 2b**)**. After the amine modification, bands for primary amino groups appeared at 3350 and 3290 cm^–1^ for NH_2_ asymmetric and symmetric stretching vibrations (superimposed on the broad OH band) and distinct increase of the band intensities of CH_2_ groups observed at 2924 and 2867 cm^–1^. These bands confirmed the presence of the (silyl)­propylamines. The increased intensity of CH_3_ bands, especially the band at 2955 cm^–1^ for asymmetric stretching, clearly showed the presence of the methyl silane. In the complex fingerprint region, the band for the NH_2_ bending mode still can be observed at 1590 cm^–1^. However, the possible formed carbamate bands caused by the adsorbed CO_2_ from air make this region even more complicated.

**Figure 2:**
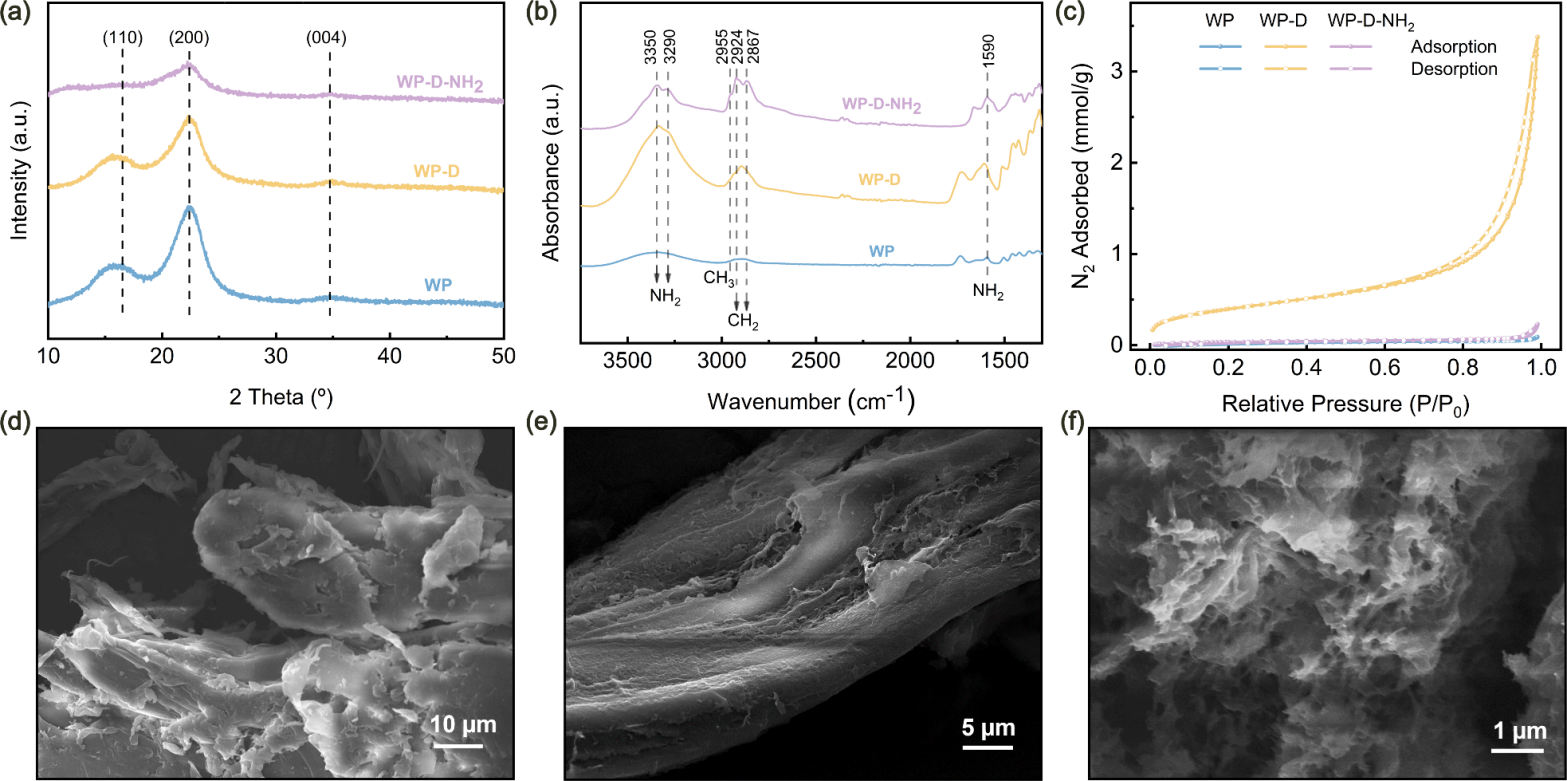
Structural and morphological characterization of WP, WP-D, and WP-D-NH_2_: (a) XRD patterns, (b) IR spectra, and (c) N_2_ adsorption–desorption isotherms; SEM images of (d) WP, (e) WP-D, and (f) WP-D-NH_2_.

Porosity plays a key role in gas adsorption by facilitating diffusion. Analysis of N_2_ adsorption–desorption isotherms (Figure 2c) showed that pristine WP had a low surface area of 2 m^2^/g (Table S2). The surface area of WP-D increased slightly to 32 m^2^/g owing to structural disruption and pore formation. However, after amination, the surface area of WP-D-NH_2_ drops to 3 m^2^/g, because the introduced aminated organosilanes partially occupied or blocked the pores, reducing effectively the porosity.^42^ SEM images (Figure 2d–f) further illustrate the morphological evolution. Pristine WP (Figure 2d) has a dense, compact structure with smooth surfaces and irregular flake-like particles, which is typical of natural lignocellulosic materials with low porosities. The structure of WP-D (Figure 2e) became more fragmented with layered and rough surfaces, indicating the defibrillation of cellulose. This structural loosening increased the surface area and provided more reactive sites for subsequent modification. After amination (Figure 2f), the adsorbent retained some porous features; however, the structure became, overall, more collapsed and compact. These changes correlated with the reduced surface area on amination, as described above. Nevertheless, the increased presence of amino groups enhanced CO_2_ chemisorption capacity, as confirmed by subsequent adsorption measurements.

Optical properties of WP, WP-D, and WP-D-NH_2_ were evaluated using UV–vis spectroscopy over the wavelength range 200–800 nm. As the samples were opaque (Figure S2), their transmittance spectra (Figure 3a) remained near 0% across the entire measured wavelength range, suggesting that their optical behavior was dominated by reflection and absorption. The reflectance (Figure 3b) increased with the wavelength for WP, reaching nearly 90% at 800 nm. In contrast, WP-D showed consistently low reflectance (<10%) across all wavelengths, indicating enhanced light absorption, due to the preservation of *in situ* regenerated lignin. WP-D-NH_2_ exhibited an intermediate reflectance, suggesting that the introduction of aminated organosilanes partially restored the reflectivity. Correspondingly, the light absorption of WP (Figure 3c) decreased from close to 100% at 200 nm to ∼20% at 800 nm, indicating strong UV absorption and weak absorption in the visible region. WP-D maintained high absorption (>90%) throughout the entire studied range, which was attributed to the deconstruction of natural wood and increased surface roughness. WP-D-NH_2_ retained intermediate absorption with a moderate decline at higher wavelengths, owing to functionalization with aminated organosilanes. To evaluate the photothermal performance, the surface temperature changes of the samples under simulated sunlight were recorded using an infrared camera (Figure 3d–f**)**. In the absence of illumination, all samples were maintained at ambient temperature (∼23 °C) (Figure 3d). Upon exposure to 1 sun irradiation for 5 min, WP-D exhibited the highest surface temperature increase, reaching 48.5 °C, followed by WP-D-NH_2_ at 45.0 °C, whereas WP showed the lowest increase at 34.5 °C. The superior photothermal response of WP-D was attributed to its enhanced light absorption, whereas the slight decrease observed for WP-D-NH_2_ resulted from the surface modification. Under 2 sun irradiation, all samples experienced further temperature increases, reaching 45.5 °C (WP), 73.3 °C (WP-D), and 67.0 °C (WP-D-NH_2_), following the same trend. The significantly improved photothermal performance of WP-D under strong illumination highlighted the role of structural changes in improving solar energy conversion. Notably, the amination only slightly reduced this effect, so that the adsorbent maintained the improved photothermal performance compared to the original WP. To assess whether the photothermal effect extends beyond the surface layer, the side vertical temperature distribution (Figure S4) within WP-D-NH_2_ was investigated. IR images were captured under different illumination times under both 1 and 2 sun irradiation (Figures S5–S10). The corresponding temperature evolution (Table S3) shows that the top surface temperature rises rapidly to the target temperature in 5 min, whereas the side vertical area exhibited slightly lower temperatures. With an extended illumination time, the average temperature in the side vertical regions approaches that of the top surface. These observations indicate that the small laboratory-scale sample can be effectively heated throughout the as-synthesized sorbent. For practical applications, the larger sorbents need to be exposed to a longer irradiation time to eventually achieve uniform regeneration temperatures.

**Figure 3:**
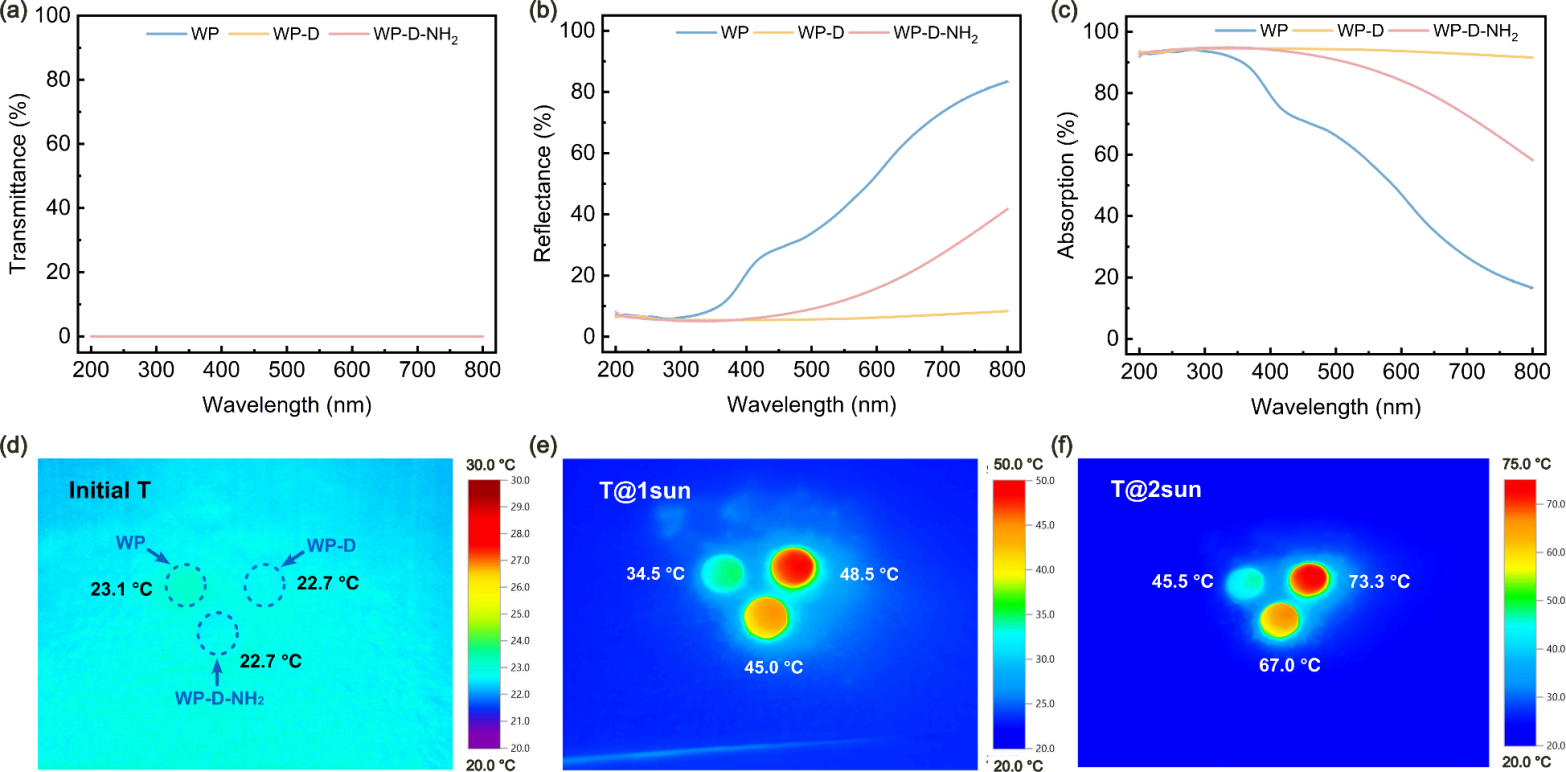
Optical and photothermal properties of WP, WP-D, and WP-D-NH_2_. (a) UV–vis transmittance spectra, (b) reflectance spectra, and (c) calculated absorption in the range of 200–800 nm. (d) Surface temperature in the absence of sun irradiation, (e) surface temperature after 5 min under 1 sun irradiation (1000 W m^–2^), and (f) surface temperature after 5 min under 2 sun irradiation (2000 W m^–2^).

Initially, we compared the CO_2_ uptake of WP, WP-D, and WP-D-NH_2_ through gas sorption isotherm measurements at 25 °C. WP and WP-D exhibited low CO_2_ uptakes of 0.20 and 0.15 mmol/g at 1 bar, respectively (Figure S11). WP-D-NH_2_ exhibited a dramatically increased CO_2_ adsorption capacity of 1.84 mmol/g (Figure 4a). This was attributed to the amination and chemisorption of CO_2_. Moreover, the sharp increase in the uptake at low pressure (Figure 4a) suggested a dense amination.^43^ At ambient CO_2_ partial pressure (∼0.4 mbar, 400 ppm) the sorbent adsorbed ∼0.2 mmol/g of CO_2_, demonstrating excellent potential for DAC. The C, H, O, and N contents of WP, WP-D, and WP-D-NH_2_ were investigated through elemental analysis (Figure S12). WP-D-NH_2_ had a nitrogen content of 4.08 wt %, and the calculated amine efficiency of 0.63 mol of CO_2_ per mol of N (Table S4) indicated the effective use of the aminosilane moieties even in dry conditions. This value exceeds the theoretical maximum amine efficiency of 0.5 mol of CO_2_ per mol of amine for carbamate formation under dry conditions, which can be attributed to two contributing effects. First, CO_2_ physisorption leads to overstoichiometric uptake,^44–46^ as evidenced by the shape of CO_2_ sorption isotherms and temperature dependence of the CO_2_ uptake (Figure 4a,b). Second, cooperative amine-silanol interactions might further enhance CO_2_ adsorption, carbamic acid can also form on single amino groups stabilized by nearby silanol groups or carbamates.^47^ Under dry conditions this carbamic acid can be condensed onto the silanol groups to form surface-bound carbamates.^48^ Theoretically, an amine efficiency of 100% is possible with the cooperative amine-silanol interaction.^49^ Temperature dependency is important to DAC processes, and the CO_2_ uptake was studied at 0, 25, and 50 °C (Figures 4b and S13). The adsorption profiles in the low-pressure region (<0.05 bar) remained nearly identical across all three temperatures, indicative of chemisorption. However, the adsorption capacity at 1 bar decreased with increasing temperature, which is normal for physisorption of CO_2_.^44^ The adsorption capacity at 0.15 bar was above 1.25 mmol/g at all of these three temperatures, which is relevant in, e.g., CO_2_ capture from flue gas. As expected, the N_2_, O_2_, and Ar adsorption capacities were low (Figure 4c). The CO_2_ gravimetric adsorption and kinetics of WP-D-NH_2_ were studied by a STA. A mass gain of 8.1 wt % was observed within 120 min (Figure S14), corresponding to a CO_2_ uptake of 1.84 mmol/g, which is consistent with volumetric measurements at 25 °C and 1 bar. The adsorbent reached 50% of its capacity within 7 min and 80% within 38 min (Figure 4d), indicating fast adsorption kinetics for CO_2_. Moisture in ambient air can strongly affect CO_2_ adsorption capacity via coadsorption of CO_2_ and H_2_O.^50^ With H_2_O, aminated adsorbents might form bicarbonate species, whereas under dry conditions, carbamates are the dominant product.^51,52^ The volumetric H_2_O adsorption of the WP-D-NH_2_ adsorbent was measured under the H_2_O saturation pressure at 25 °C (Figure 4e). The adsorption isotherm did not exhibit the typical shape of a hydrophilic material. Its H_2_O sorption was basically following Henry’s law until relative pressure of 0.6, beyond which capillary condensation likely occurred, indicating a moderate hydrophilicity of WP-D-NH_2_. Because it has hydrophilic amine groups and cellulose^53^ and hydrophobic lignin moieties. Additionally, the H_2_O uptake of WP-D-NH_2_ at a series of relative humidity (RH) condition at 25 °C was also investigated (Figure S15). This relatively low H_2_O uptake until RH = 60% has positive implications for DAC processes as less hygroscopic adsorbents implies less latent heat during desorption.^54^

**Figure 4:**
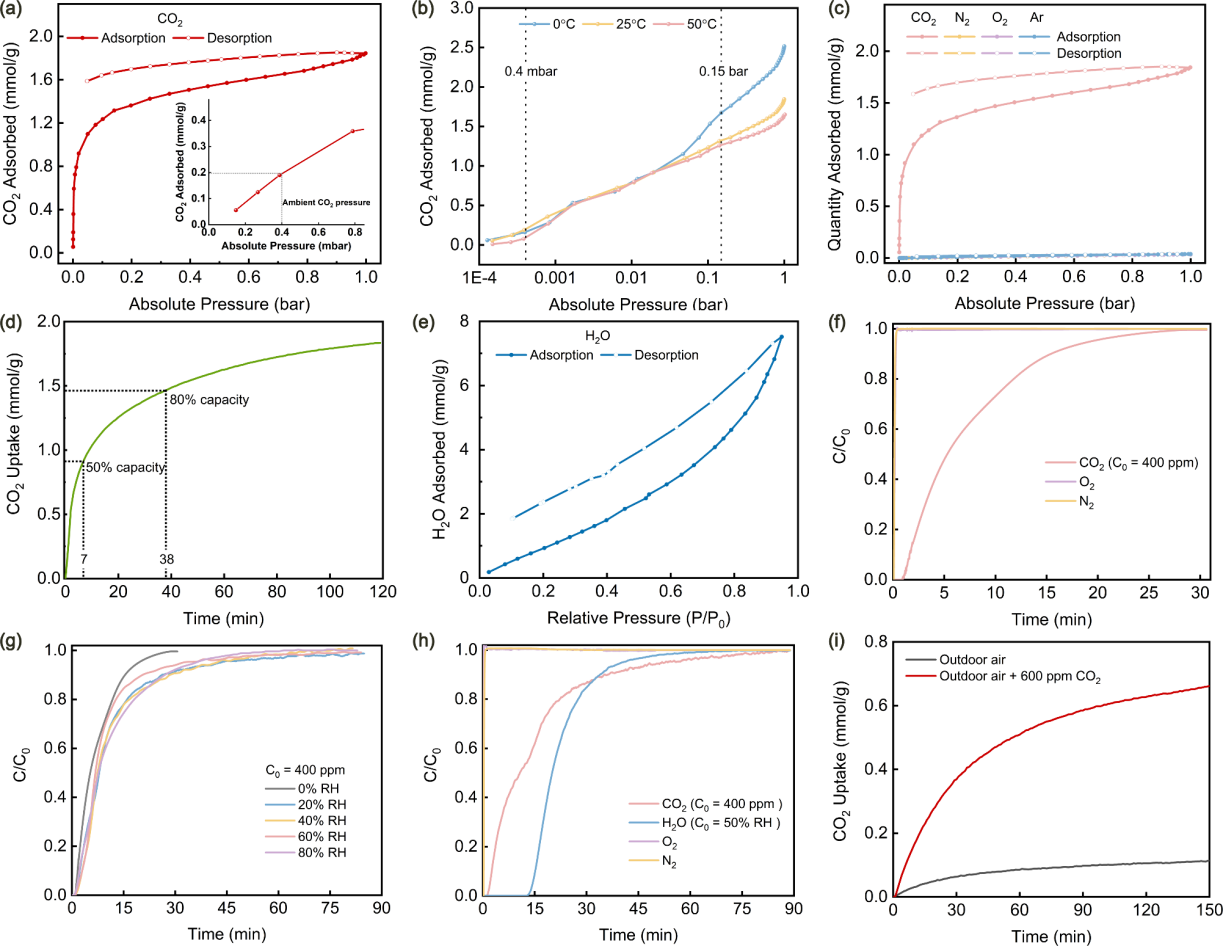
Gas sorption capacity and kinetics of WP-D-NH_2_. (a) CO_2_ sorption isotherm measured at 25 °C and the inset zooms in to the region of ambient CO_2_ pressure (0.4 mbar). (b) CO_2_ adsorption isotherms measured at 0, 25, and 50 °C. (c) Comparison of single-component CO_2_, N_2_, O_2_, and Ar sorption isotherms measured at 25 °C. (d) CO_2_ adsorption kinetics at 25 °C. (e) H_2_O sorption isotherm measured at 25 °C. (f) CO_2_, O_2_, and N_2_ breakthrough curves under simulated dry air at 25 °C. (g) CO_2_ breakthrough curves under simulated air with different relative humidity values at 25 °C. (h) CO_2_, H_2_O, O_2_, and N_2_ breakthrough curves under simulated air (400 ppm of CO_2_ at 50% RH) at 10 °C. (i) CO_2_ uptake at 25 °C from outdoor open air in Stockholm, Sweden, and concentrated air (outdoor air with 600 ppm of CO_2_). *C* and *C*_0_ represent the concentrations in the inlet and outlet gas flow, respectively.

The CO_2_ uptake performance of the adsorbent under atmospheric conditions was evaluated by using breakthrough experiments. The breakthrough time (*C*/*C*_0_ ≈ 0.05–0.1) and saturation time (*C*/*C*_0_ ≈ 0.95–1.0) provided quantitative insights into the adsorption capacity and kinetics. The adsorption behavior under simulated dry air (400 ppm of CO_2_ in a mixture of O_2_/N_2_ with a volume ratio of 1:4) at 25 °C (Figure 4f) showed a significant uptake of CO_2_ by the WP-D-NH_2_ adsorbent. This was evidenced by delayed breakthrough times for CO_2_, while N_2_ and O_2_ passed through the column without adsorption (*C*/*C*_0_ = 1), consistent with a high selectivity of the adsorbent toward CO_2_. Under different RH values at 25 °C (Figure 4g), the breakthrough occurred slightly earlier under dry condition but was delayed under humid conditions, showing that presence of water vapor increases the CO_2_ capacity of the adsorbent and it is advantageous for CO_2_ adsorption. All breakthrough curves under humid conditions showed similar behavior near saturation, indicating that the WP-D-NH_2_ adsorbent maintained an effective CO_2_ capture performance across a wide range of RH values. From breakthrough curves, the resulting CO_2_ adsorption capacity in the presence of water vapor is higher than the capacity under dry condition (Figure S16). The H_2_O uptake increased at higher RH (Figure S16 and cf. Figure 4e), consistent with the moderately hydrophilic nature of WP-D-NH_2_. In addition to measurements at 25 °C, we monitored the local climate in Stockholm, Sweden, over a two-week period (Figures S17–S19). The average temperature, RH, and CO_2_ concentration were 9 °C, 51%, and 415 ppm, respectively (Table S5). Based on these conditions, the adsorption behavior was further evaluated under simulated air with 50% RH at 10 °C (Figure 4h). CO_2_ began breaking through after approximately 10 min and reached saturation around 40 min, while H_2_O showed a longer breakthrough delay, saturating closer to 60 min due to the moderately hydrophilic WP-D-NH_2_ adsorbent. CO_2_ breakthrough occurred prior to full saturation with H_2_O, indicating that H_2_O did not compete with CO_2_ for available sites. Finally, we evaluated CO_2_ capture performance with open air in Stockholm, Sweden (Figure 4i). When exposed to outdoor ambient air (∼400 ppm of CO_2_), the adsorbent captured 0.15 mmol/g of CO_2_ at 25 °C. Upon introducing 600 ppm of CO_2_ to make a “concentrated air”, the CO_2_ uptake increased significantly. This improvement shows its potential for indoor CO_2_ capture application, where CO_2_ concentration is typically higher than 1000 ppm and may cause concerns about air quality.^55^

Thermal desorption of CO_2_ is typically needed in DAC processes; the adsorbent WP-D-NH_2_ was stable without degradation under 200 °C (Figure S20). Hence, DSC was used to assess the heat flow with a CO_2_-saturated WP-D-NH_2_ adsorbent during the CO_2_ gravimetric adsorption experiments (Figure 5a**)**. The adsorbent was heated under a constant nitrogen flow of N_2_. A distinct endothermic peak was observed at approximately 94 °C, indicating the desorption of chemisorbed CO_2_. To investigate the temperature-dependent desorption kinetics, time-resolved CO_2_ desorption experiments were performed at 80 and 100 °C (Figure 5b). At both temperatures, approximately 50% of the adsorbed CO_2_ was released within 14 min but the desorption proceeded slightly more rapidly at 100 °C, achieving 80% desorption within 17 min, compared to 21.1 min at 80 °C (Table S6). Given that the WP-D-NH_2_ adsorbent can reach up to 45 and 67 °C under simulated solar irradiation of 1 sun and 2 sun, respectively, we further assessed its desorption performance at these temperatures to simulate solar-powered regeneration in DAC processes. At 67 °C (Figure 5c), 50% of the CO_2_ was desorbed within 22 min, while 80% desorption required 117 min, demonstrating that mild heat from solar energy is sufficient to partly regenerate the adsorbent without the need for external heating. At 45 °C (Figure S21), 53 min was needed to desorb 50% of the CO_2_, further highlighting the potential of solar-thermal regeneration. Additionally, the CO_2_ release profile at 67 °C after the CO_2_ uptake from simulated air (Figure 5d) showed that the electrical signal of the CO_2_ concentration rapidly rose to a maximum at ∼10 min, followed by a progressive decline to baseline. The sharp peak indicated fast initiation of thermally driven CO_2_ release. We further investigated the cyclic stability of WP-D-NH_2_ over multiple adsorption–desorption cycles to assess its long-term performance (Figure 5e). After 40 consecutive adsorption–desorption cycles, the WP-D-NH_2_ adsorbent maintained a stable CO_2_ uptake capacity with negligible loss, indicating the resistance of the adsorbent toward repeated thermal regeneration. The IR spectrum of WP-D-NH_2_ before and after CO_2_ capture (Figure S22) showed that the adsorbent adsorbs CO_2_ in the form of carbamates. Since the sample adsorbs CO_2_ from ambient air, it is not possible to measure the IR spectrum with the *ex situ* method (e.g., in air) without the presence of carbamates. The band at 1567 cm^–1^ corresponded to (N)­COO^–^ asymmetric stretching, and the band at 1473 cm^–1^ was assigned to the symmetric NH_3_^+^ deformation in propylammonium propylcarbamate ion pairs.^47,56^ Increase of the intensity of these bands after the CO_2_ uptake, along with the decrease of the intensity of these bands following CO_2_ desorption, confirmed the reversible formation and cleavage of CO_2_–amine bonds. This observation further demonstrated the regenerability of the adsorbent WP-D-NH_2_.

**Figure 5:**
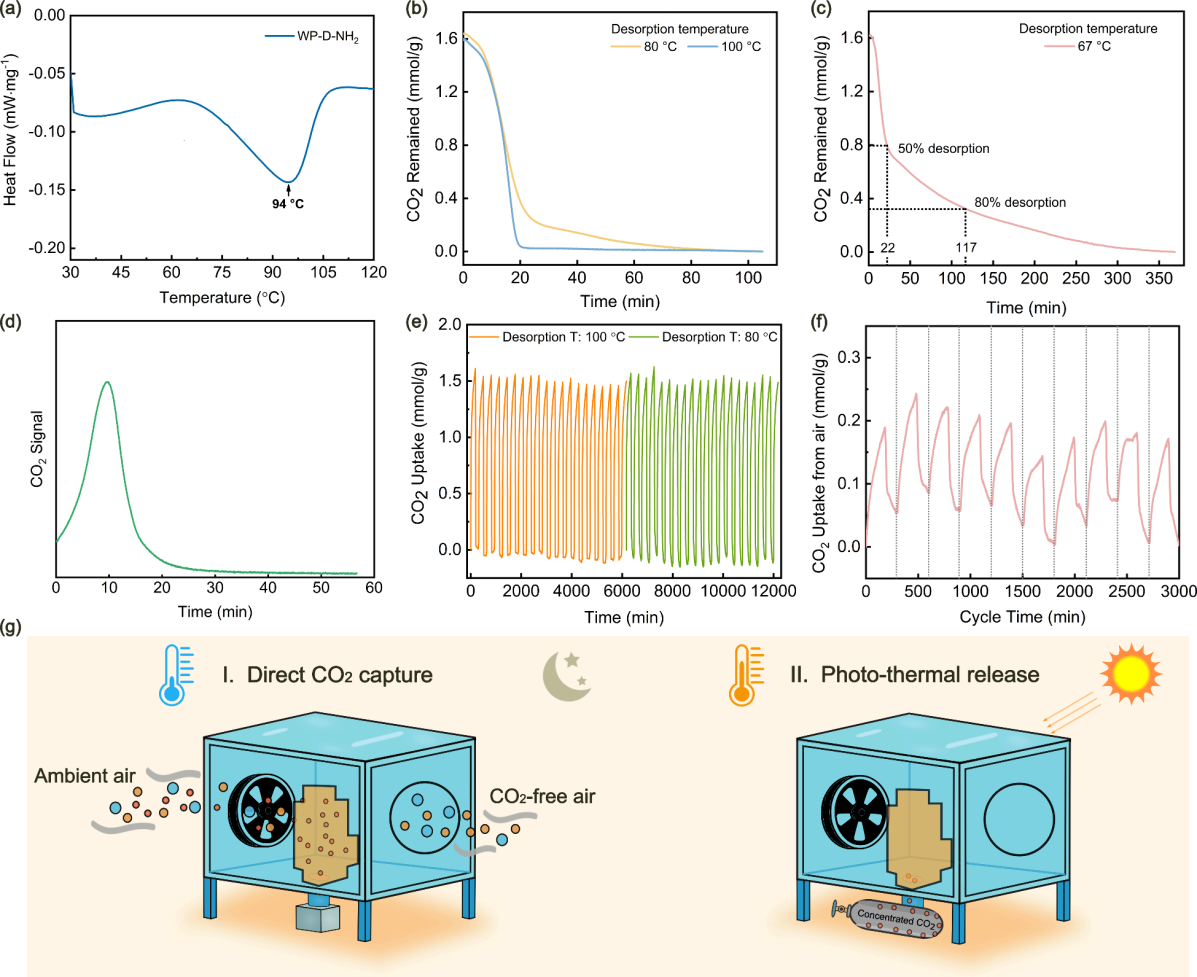
Desorption kinetics and solar-thermal regeneration demonstration of WP-D-NH_2_. (a) DSC curve. (b) Desorption at 80 and 100 °C. (c) Solar-thermal regeneration potential at 67 °C. (d) Electrical signal of CO_2_ concentration over time at 67 °C after CO_2_ capture from simulated air. (e) 40 consecutive capture–release cycles of WP-D-NH_2_ with pure CO_2_ (adsorption at 25 °C). (f) 10 consecutive capture–release cycles with outdoor ambient air. (g) Practical application installation of WP-D-NH_2_ in a DAC process.

In addition to measurements with pure CO_2_, we also assessed the performance of WP-D-NH_2_ under realistic conditions by capturing CO_2_ directly from ambient air (∼400 ppm of CO_2_). WP-D-NH_2_ exhibited a stable performance over 10 consecutive capture–desorption cycles, maintaining an adsorption capacity of approximately 0.2 mmol/g (Figure 5f). The results for WP-D-NH_2_ were compared with those of recently published studies on photothermal CO_2_ adsorbent composites for DAC, and it exhibited comparable performance (Table S7). A conceptual application scenario was proposed to illustrate the practical applicability of WP-D-NH_2_ in a DAC process (Figure 5g). It involves the use of natural temperature variations, where CO_2_ is captured from the air during the cooler nighttime period and released during the daytime through solar heating. This simple day–night cycling takes advantage of ambient thermal fluctuations, enabling energy-efficient, solar-driven regeneration of the adsorbent. However, reliance on only solar irradiation will reduce the achievable cycle frequency and overall productivity. Nevertheless, the developed sorbent with rapid adsorption–desorption kinetics can be flexibly integrated into practical DAC systems. Intermittency associated with solar irradiation can be mitigated through hybrid regeneration using waste heat or electrical heating during night times. This integration allows for continuous operation while enabling solar-assisted regeneration for energy-saving DAC process. Finally, preliminary cost evaluation (Table S8) indicates an estimated preparation cost of 314 USD per kg of dry adsorbent WP-D-NH_2_, and APDMS and freeze-drying are dominant cost contributors. However, strategies including recycling DES, using green electricity, and scalable drying can help reduce the cost in large-scale production. The cost comparison between WP-D-NH_2_, Lewatit VP OC 1065, and PEI(50)/SBA-15 (Table S9) showed that the relatively inexpensive and abundant biomass as feedstock could enhance the economic competitiveness of WP-D-NH_2_. These findings highlight the potential of WP-D-NH_2_ as a photothermal CO_2_ adsorbent containing a large fraction of waste biomass for low-energy, sustainable CO_2_ capture for DAC and potentially also concentrated gas streams with CO_2_.

## Conclusion

In this work, a new amine-functionalized CO_2_ adsorbent (WP-D-NH_2_) with solar-thermal regeneration capability was developed from wood waste. This design innovatively combined the natural photothermal properties of lignin with the chemical reactivity of cellulose to facilitate effective amination. The performance of the CO_2_ adsorbent for DAC was evaluated based on several key properties, including working capacity, selectivity, adsorption–desorption kinetics, humidity resistance, and CO_2_ uptake with simulated air and outdoor open air, photothermal regeneration under solar irradiations, and cycling stability. The resulting adsorbent achieves efficient CO_2_ capture (1.84 mmol/g at 25 °C), fast adsorption–desorption kinetics (uptake 50% of its capacity within 7 min and release 50% of CO_2_ in 22 min), photothermal desorption at 67 °C under solar illumination, high selectivity over N_2_, O_2_, and Ar, and stable performance over multiple adsorption–desorption cycles. The study also demonstrated that water vapor positively increases the CO_2_ adsorption capacity of the adsorbent, thereby improving its performance under humid air conditions. Overall, this work presented a promising strategy for developing photothermal CO_2_ adsorbents containing a large fraction of waste biomass. This highlights the critical role of renewable solar energy in enabling energy-saving DAC processes and advancing climate-resilient carbon removal technologies.

## Supplementary Material



## Notes

More detailed characterization of the samples (PDF)
