# Supplementary material for: Upcycling Wood Waste into Solar-Driven Regenerative Sorbent for Direct Air Capture
Source: ACS Sustain Chem Eng. 2026 Mar 9;14(11):5503–12. doi: 10.1021/acssuschemeng.5c12124 (PMC13014533; doi:10.1021/acssuschemeng.5c12124)
Supplement: Supplementary file 1 [file sc5c12124_si_001.pdf]

Upcycling Wood Waste into Solar-driven Regenerative Sorbent for Direct Air Capture

Man Qi[a], *Bo Pang*[b], *Aji P.Mathew*[a], *Zoltán Bacsik*[c], *Niklas Hedin*[a], *Jiayin Yuan*[a]*

[a] Department of Chemistry, Stockholm University, Stockholm 10691, Sweden.

[b] Department of Food Science and Technology, National University of Singapore, Science Drive 2, Singapore 117542, Singapore.

[c] Institute of Chemistry, University of Miskolc, 3515 Miskolc, Hungary

Corresponding email: jiayin.yuan@su.se

Table of Contents

Number of pages in the supporting information: 20

Number of figures in the supporting information: 22

Number of tables in the supporting information: 9

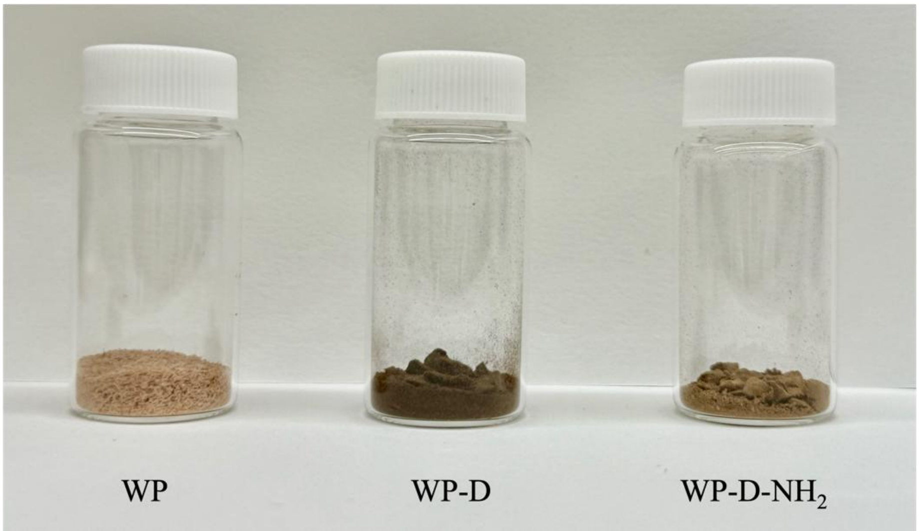

Figure S1. The photographs of samples of WP, WP-D and WP-D-NH_2 in a powdered form.

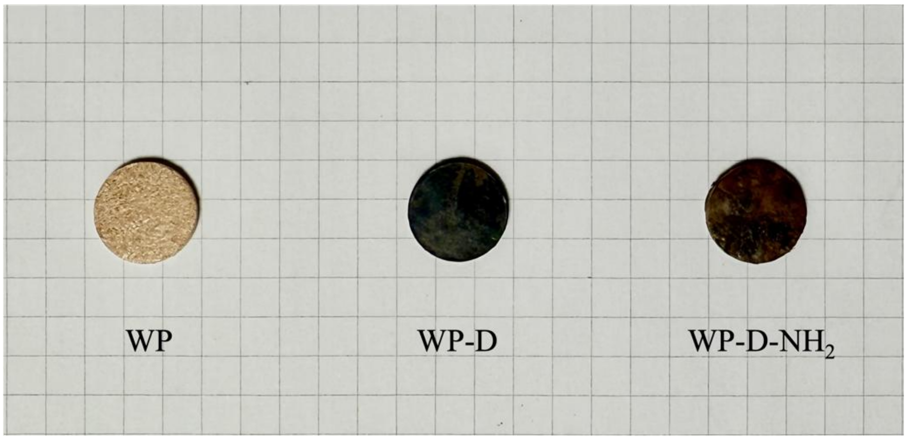

Figure S2. The photographs of samples of WP, WP-D and WP-D-NH_2 in pellet (diameter = 13 mm) for testing optical and photothermal properties.

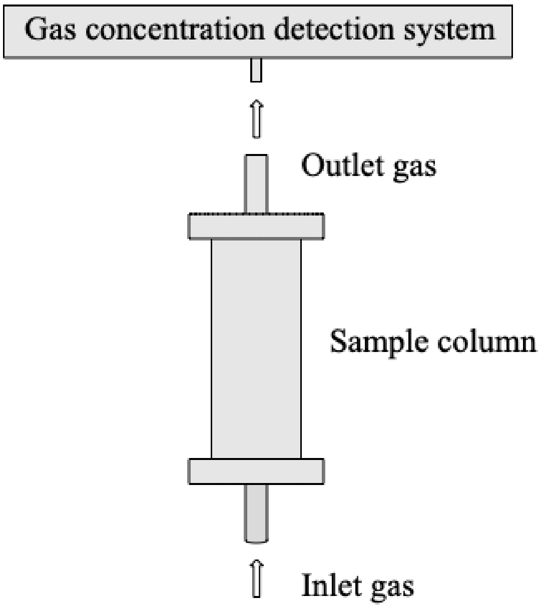

Figure S3. The experimental setup of the BSD-MAB multi-constituent adsorption breakthrough curve analyzer.

Table S1. The chemical composition of WP and WP-D.

Sample	Cellulose (%)	Hemicellulose (%)	Lignin (%)
WP	50.40	21.89	19.97
WP-D	22.91	6.62	33.80

Data provided are the average of two measurements.

Table S2. Measurements of specific surface area by the BET (Brunauer-Emmett-Teller) equation and average pore size by the BJH (Barrett-Joyner-Halenda) method for the samples.

Sample	BET specific surface area (m^2/g)	Average pore size by BJH method (nm)	Total pore volume (cm^3/g)
WP	2	8.6	-
WP-D	32	18.2	-
WP-D-NH_2	3	16.1	-

Data provided are the average of three measurements.

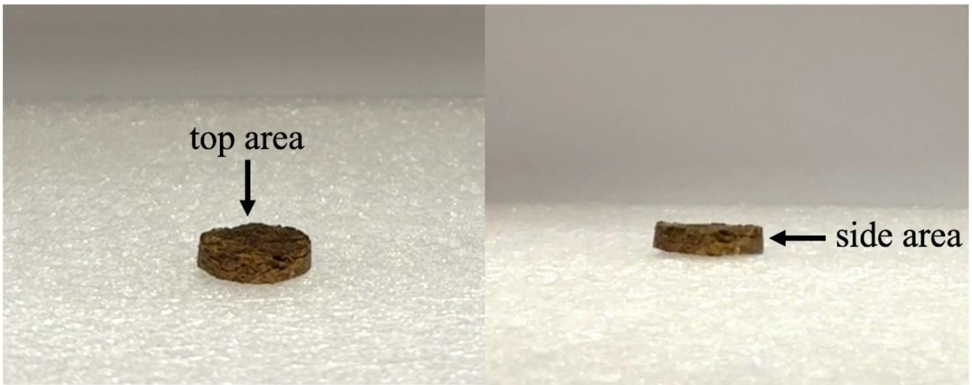

Figure S4. IR camera measurement locations during solar irradiation on the sorbent of WP-D-NH_2 including the top surface (left image) and the side vertical area (right image).

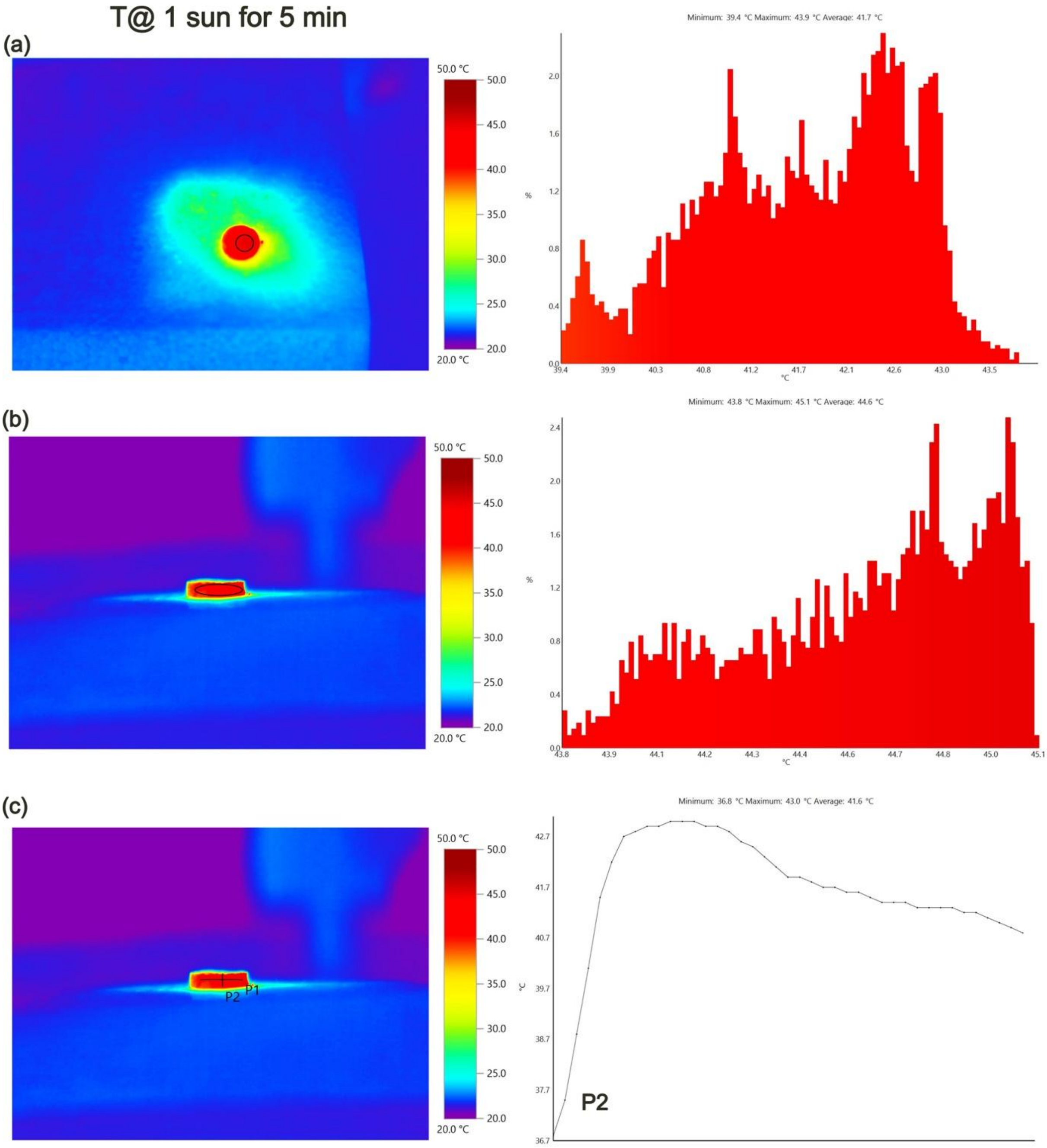

Figure S5. Temperature profiles of WP-D-NH_2 at the top surface (a) and two side vertical areas (b and c) under 1 sun (1000 W m^{-2}) for 5 min.

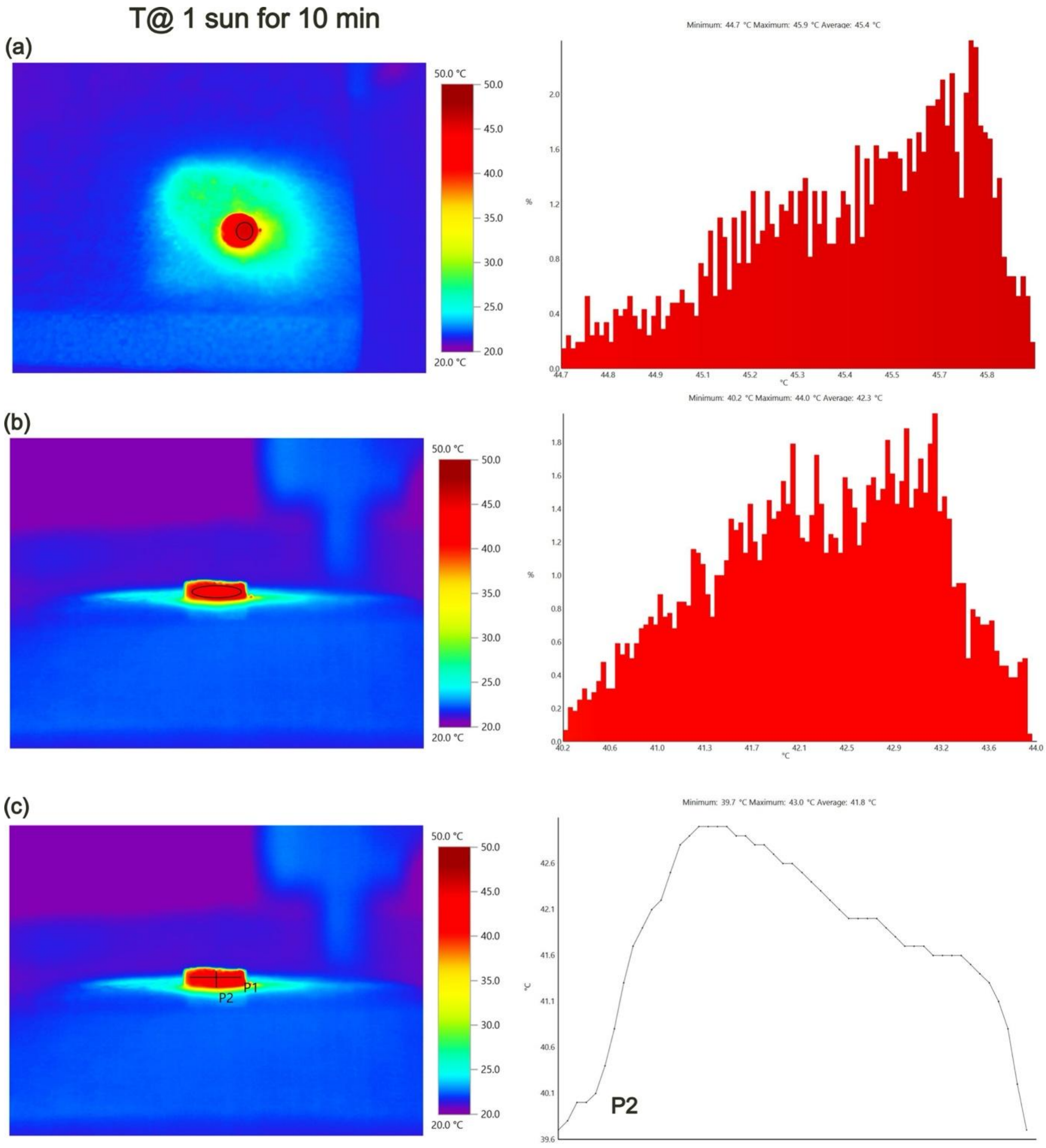

Figure S6. Temperature profiles of WP-D-NH_2 at the top surface (a) and two side vertical areas (b and c) under 1 sun (1000 W m^{-2}) for 10 min.

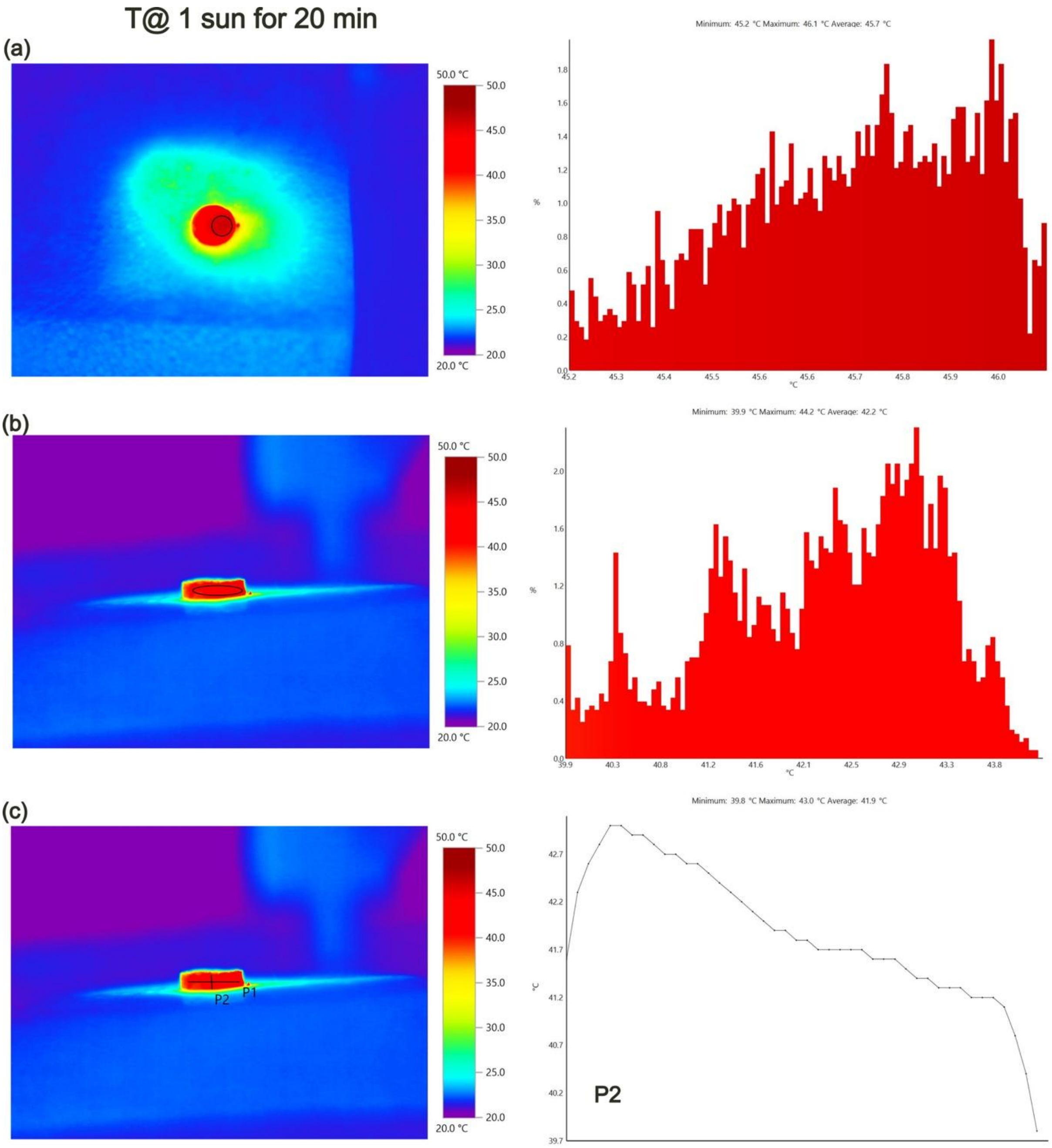

Figure S7. Temperature profiles of WP-D-NH_2 at the top surface (a) and two side vertical areas (b and c) under 1 sun (1000 W m^{-2}) for 20 min.

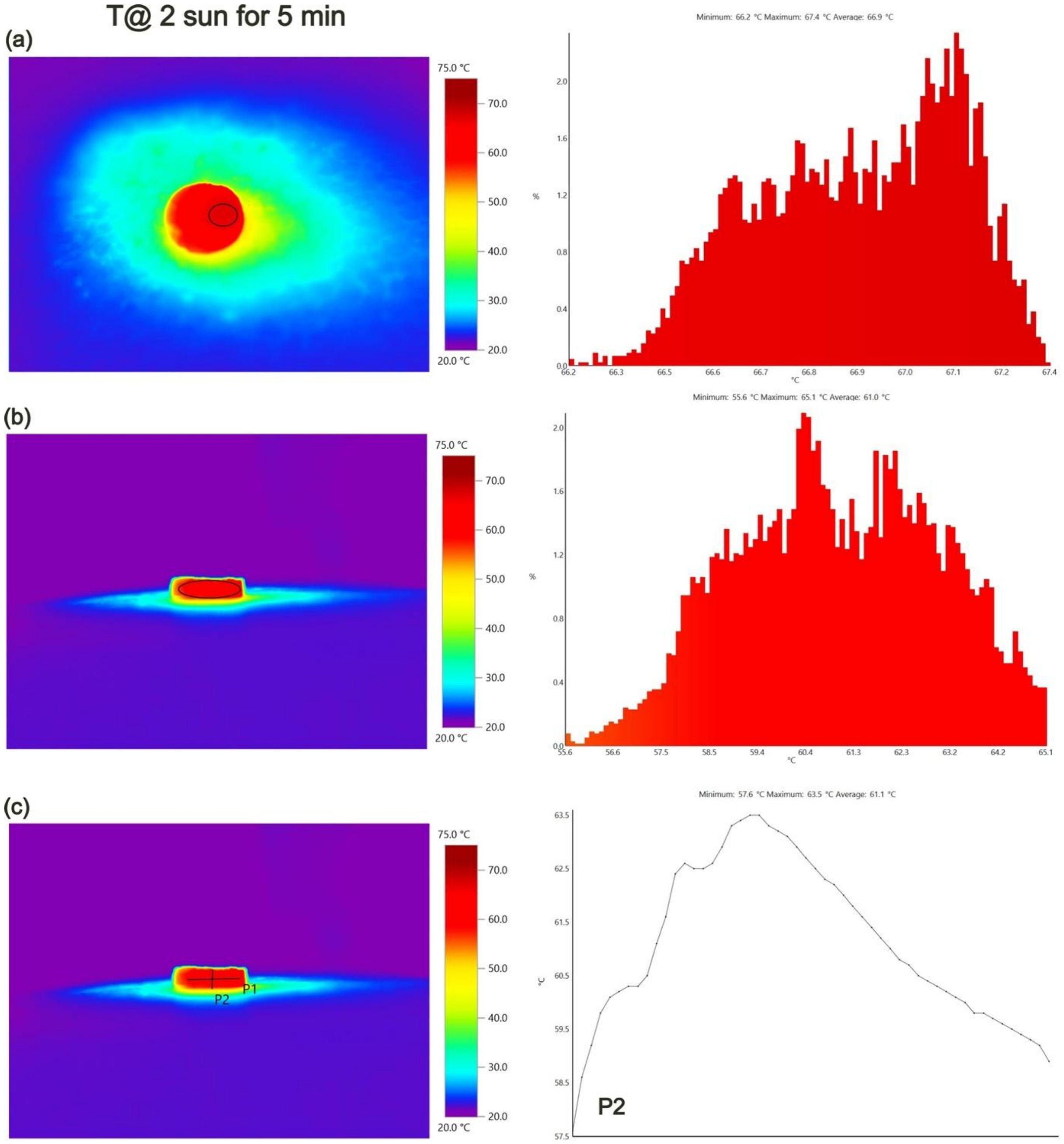

Figure S8. Temperature profiles of WP-D-NH_2 at the top surface (a) and two side vertical areas (b and c) under 2 sun (2000 W m^{-2}) for 5 min.

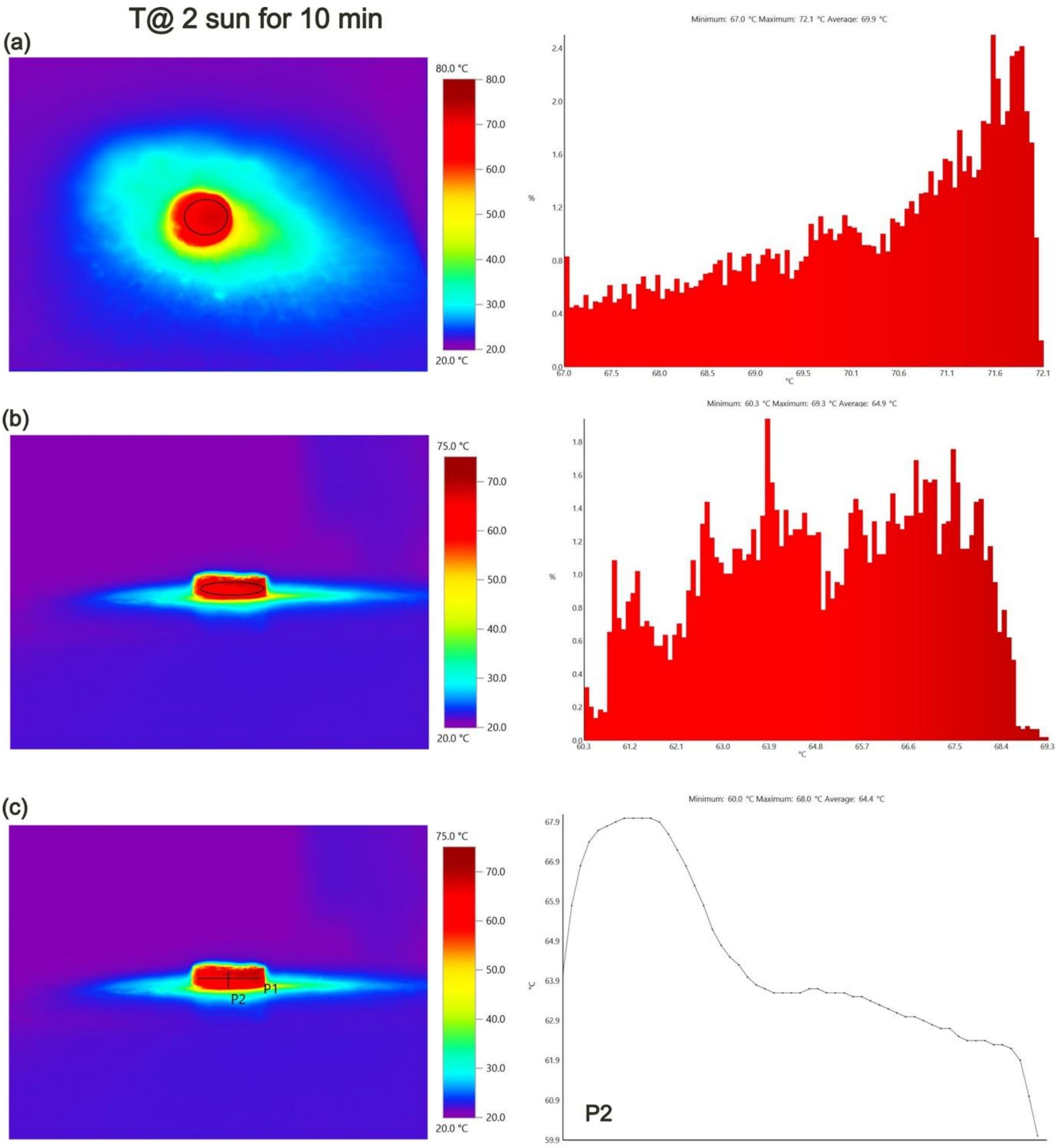

Figure S9. Temperature profiles of WP-D-NH_2 at the top surface (a) and two side vertical areas (b and c) under 2 sun (2000 W m^{-2}) for 10 min.

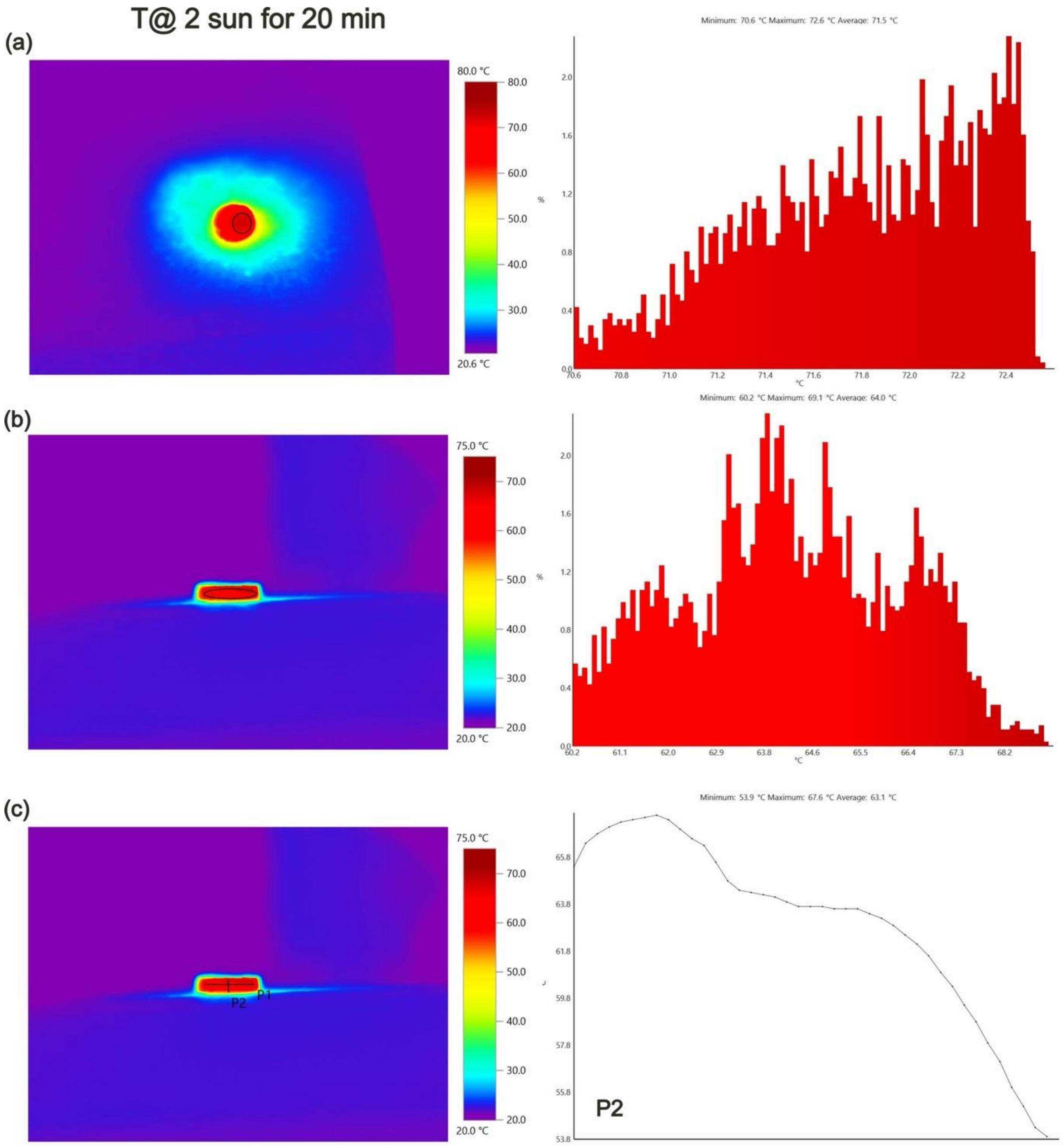

Figure S10. Temperature profiles of WP-D-NH_2 at the top surface (a) and two side vertical areas (b and c) under 2 sun (2000 W m^{-2}) for 20 min.

Table S3. Temperature evolution of WP-D-NH_2 at the top surface and the side vertical areas under 1 sun (1000 W m^{-2}) and 2 sun (2000 W m^{-2}) irradiation.

**Temperature without sun irradiation (°C)**						
Time (min)	top area			side area		
	minimum	maximum	average	minimum	maximum	average
0	23.2	23.6	23.4	22.3	22.7	22.5
**Temperature under 1 sun irradiation (°C)**						
Time (min)	top area			side area		
	minimum	maximum	average	minimum	maximum	average
5	43.8	45.1	44.6	39.4	43.9	41.7
10	44.7	45.9	45.4	40.2	44.0	42.3
15	44.5	45.7	45.2	40.0	43.7	42.2
20	45.2	46.1	45.7	39.9	44.2	42.2
**Temperature under 2 sun irradiation (°C)**						
Time (min)	top area			side area		
	minimum	maximum	average	minimum	maximum	average
5	66.2	67.4	66.9	55.6	65.1	61.0
10	67.0	72.1	69.9	60.3	69.3	64.9
15	69.4	71.4	70.5	59.8	67.8	63.7
20	70.6	72.6	71.5	60.2	69.1	64.0

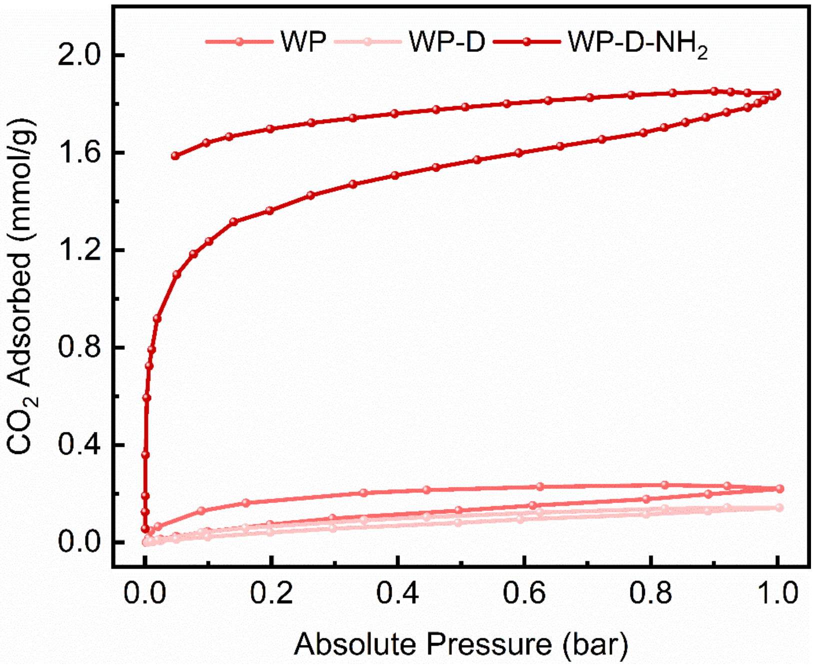

Figure S11. CO_2 sorption isotherms of samples for WP, WP-D and WP-D-NH_2 at 25 °C.

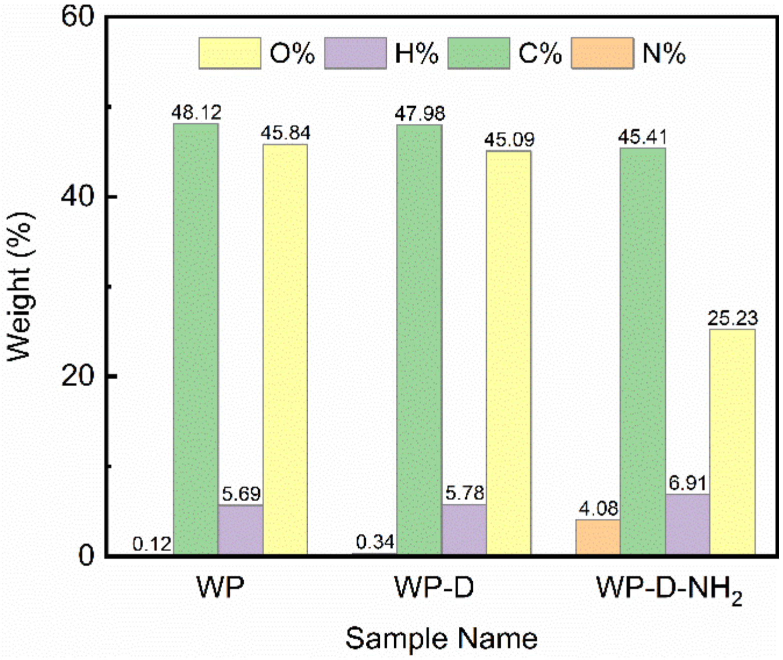

Figure S12. C, H, O and N mass contents in samples of WP, WP-D and WP-D-NH_2.

Table S4. Table of amine efficiency derivation. (Q_{CO2} measured at 25 °C and 1 bar)

Sample	N (wt%)	The mol of N (mmol g^{-1})	Q_{CO2} (mmol g^{-1})	Amine efficiency (mol-CO_2 mol^{-1}-N)
WP-D-NH_2	4.08	2.91	1.84	0.63

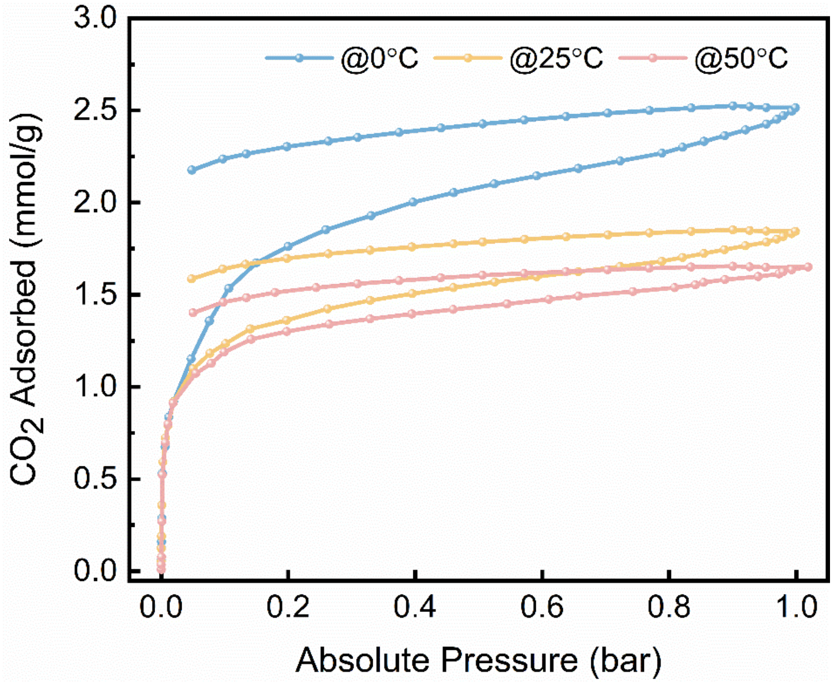

Figure S13. CO_2 full sorption isotherms of WP-D-NH_2 measured at 0 °C, 25 °C, and 50 °C.

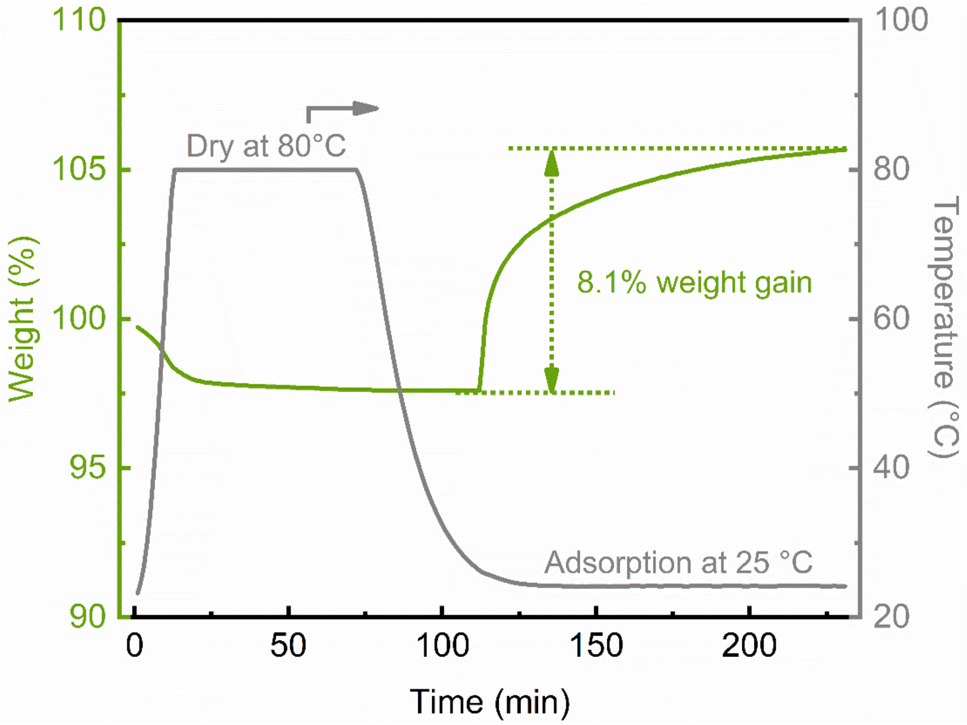

Figure S14. STA temperature profile and CO_2 gravimetric adsorption of WP-D-NH_2.

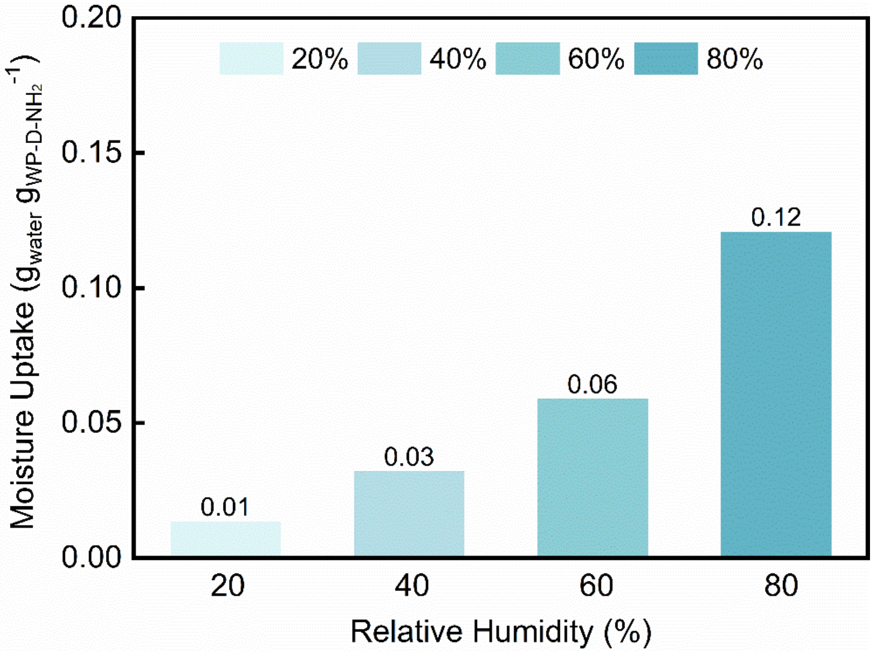

Figure S15. Moisture uptake measured at 25 ℃ for 4h under different relative humidity conditions.

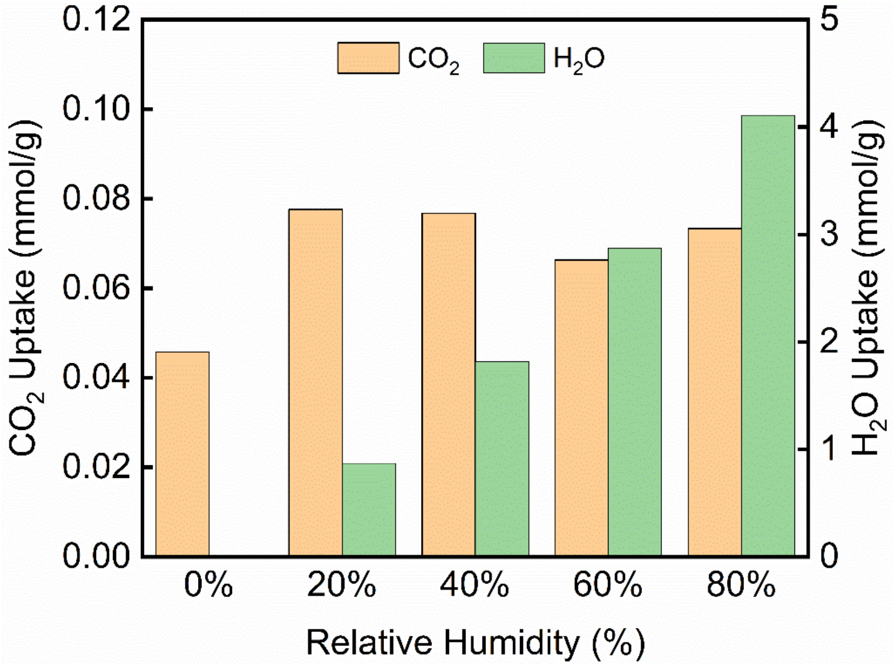

Figure S16. CO_2 and H_2O uptake under 400 ppm of CO_2 at 20%, 40%, 60% and 80% RH at 25 °C.

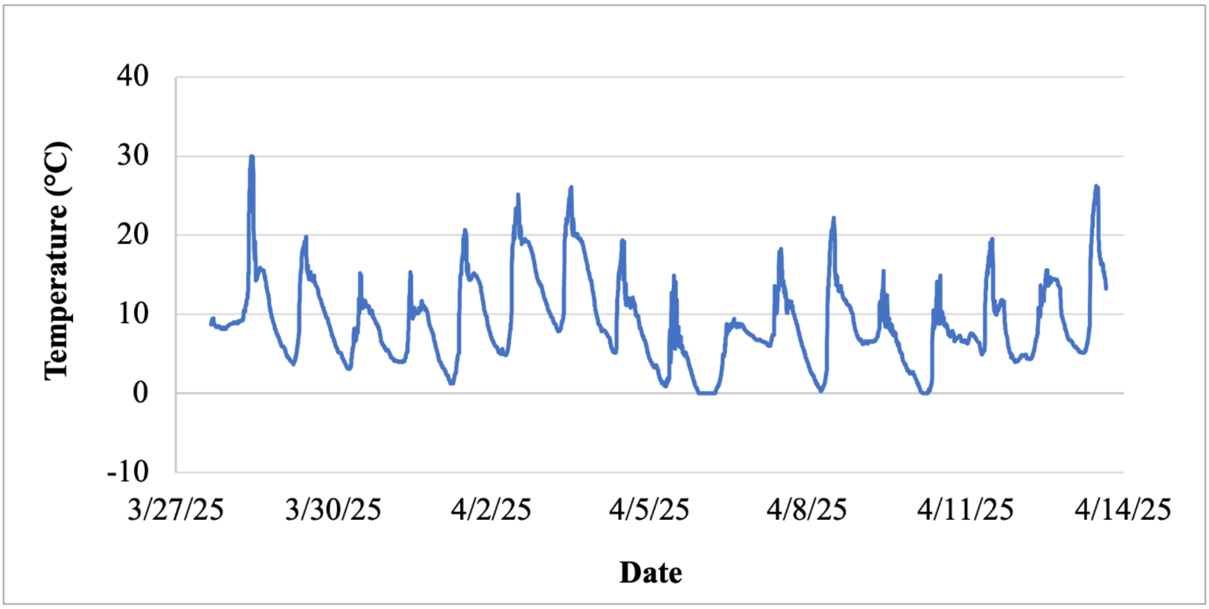

Figure S17. Ambient outdoor temperature data was monitored for 17 continuous days.

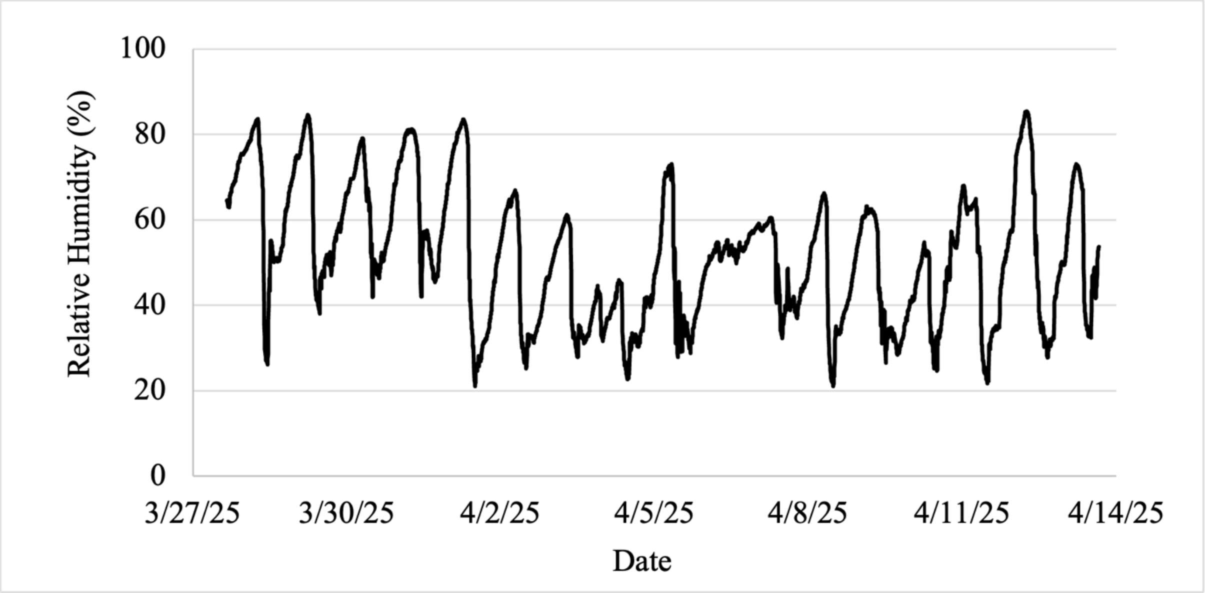

Figure S18. Ambient outdoor RH data was monitored for 17 continuous days.

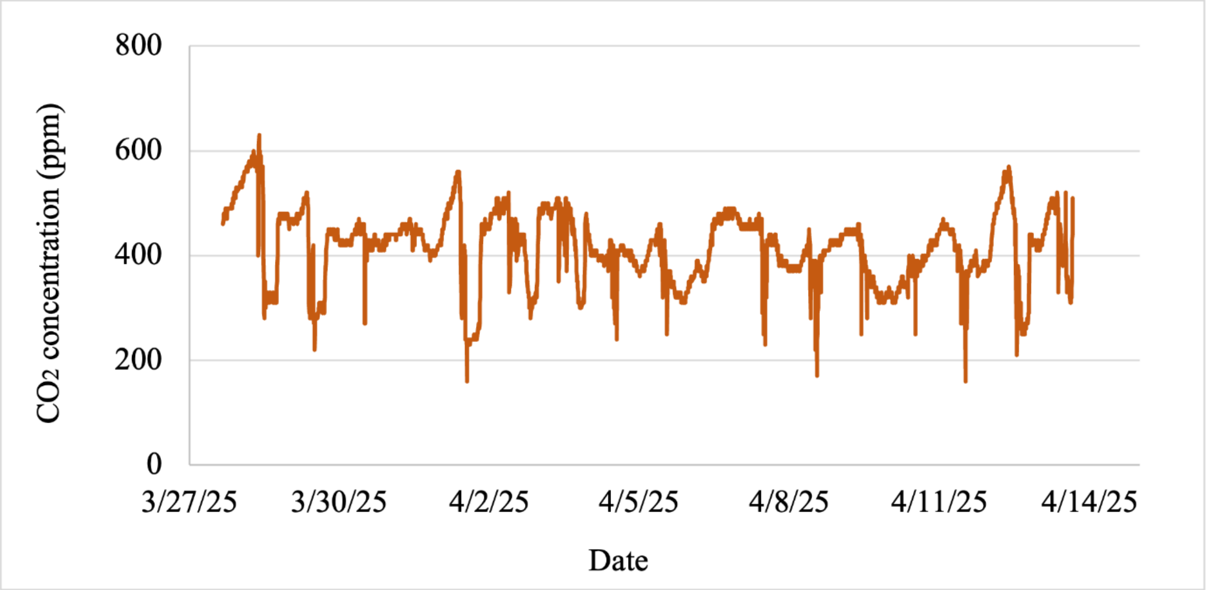

Figure S19. Ambient outdoor CO_2 concentration data was monitored for 17 continuous days.

Table S5. Average temperature, RH and CO_2 concentration for 17 continuous days.

Average temperature (°C)	Average RH (%)	Average CO_2 concentration(ppm)
9	51	416

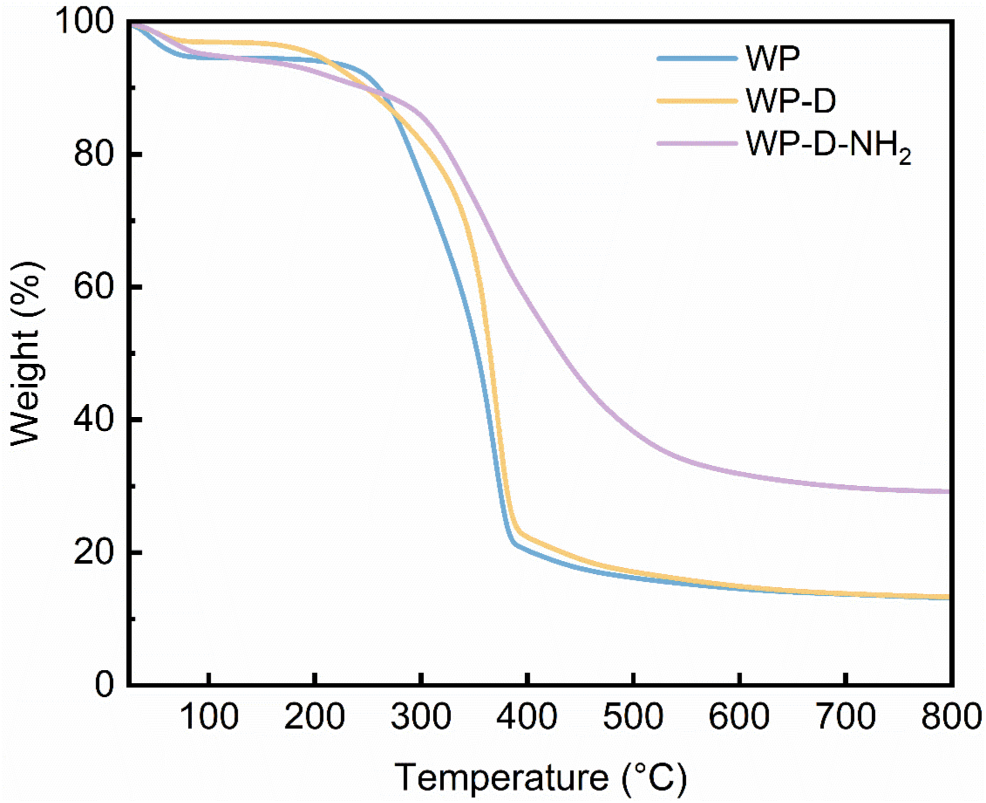

Figure S20. TGA plots of samples for WP, WP-D and WP-D-NH_2 under N_2.

Table S6. Desorption kinetics of WP-D-NH_2 measured at 80 and 100 °C.

Desorption temperature (°C)	Time of 50% CO_2 desorbed (min)	Time of 80% CO_2 desorbed (min)
80	14.8	21.1
100	14.4	17.0

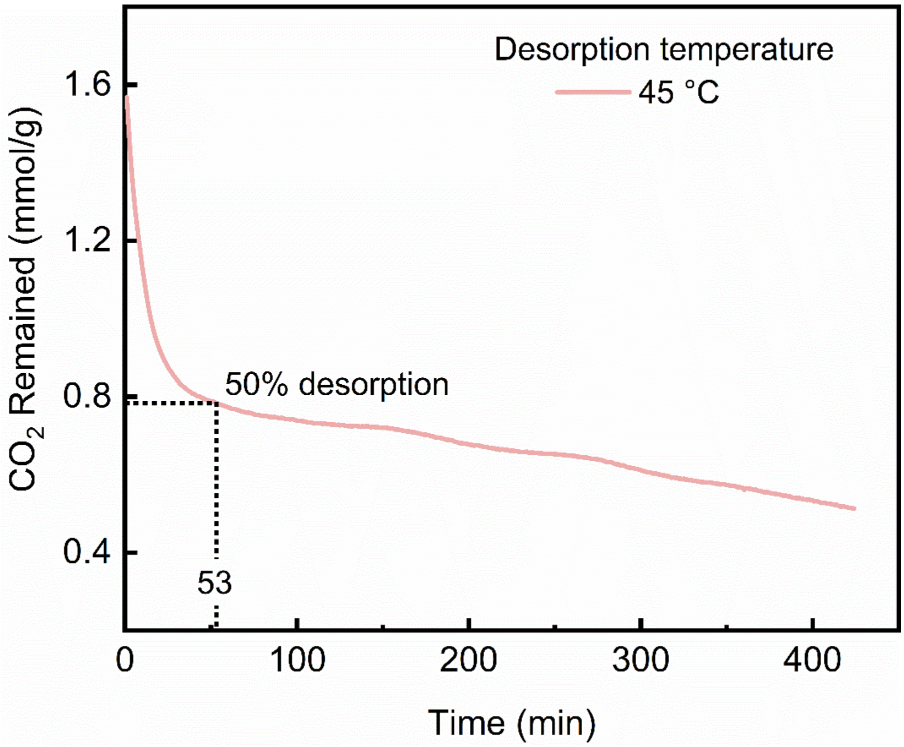

Figure S21. Solar-thermal regeneration potential of WP-D-NH_2 at 45 °C.

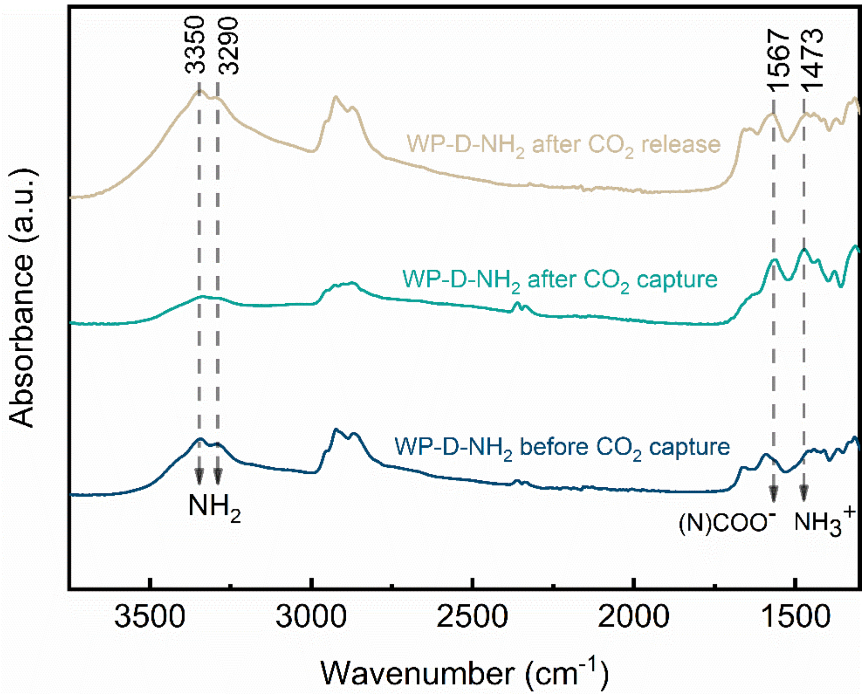

Figure S22. IR spectra of WP-D-NH_2 before and after CO_2 uptake/release.

Table S7. Comparison of this work and recent photothermal CO_2 adsorbents in literature.

Materials	CO_2 Adsorption at 25 °C (mmol/g)	CO_2 Uptake from air (mmol/g)	Photothermal desorption T (°C) under 2 sun	Ref
PEI(70)@HCS	1.31 (10% CO_2/N_2)	/	120	1
Aminosilia@MXene(7 wt%)	/	1.63 at 25 °C	76.8	2
AmSiC aerogel	/	1.01 at 25 °C	70	3
Carbon/Silica Composite	/	0.14 at 35 °C	73	4
**WP-D-NH$_2$**	**1.84 (100% CO_2)**	**0.15 at 25°C**	**67**	**this work**

Note: PEI@HCS (hollow carbon spheres encapsulating poly(ethylenimine)), AmSiC (amine-modified silica / carbon).

Table S8. Estimated cost to produce 1 kg of dry adsorbent WP-D-NH$_2$.

Component	Quantity per batch	Unit price (USD/kg)	Cost (USD/kg)	Source of bulk commodity price
Waste wood powder	3.23 g	0.10	0.06	https://www.bioboost.eu/uploads/files/bioboost_d1.1-syncom_feedstock_cost-vers_1.0-final.pdf?
Choline chloride	27.92 g	0.60	2.95	https://businessanalytiq.com/procurementanalytics/index/choline-chloride-price-index/
Oxalic acid	18.01 g	0.57	1.79	https://www.intratec.us/solutions/primary-commodity-prices/commodity/oxalic-acid-prices
tert-butanol	16.00 g	1.04	2.93	https://www.intratec.us/solutions/primary-commodity-prices/commodity/tert-butyl-alcohol-prices
APDMS	6.00 g	110.00	116.20	https://www.chemimpex.com/products/27567
Electricity (freeze drying)	20 kWh	0.05/ kWh	190.14	https://statsskuld.se/en/elpris
**Total**	/	/	314.07	/

Note: calculation is based on lab-scale batch of 5.68 g.

Table S9. Comparison of cost between WP-D-NH_2 and typical solid sorbents used in DAC.

Sorbent	Type	Preparation cost (USD/kg)	Reference
Lewatit® VP OC 1065	Commercially available	691	Sigma-Aldrich
PEI(50)/SBA-15	Lab-scale	760	[5]
WP-D-NH_2	Lab-scale	314	This work

References

(1) Reversible Adsorption and Light-Driven Release of CO_2 Using Hollow Carbon Sphere-Based Adsorbents with Photothermal Conversion Feature | ACS Sustainable Chemistry & Engineering. https://pubs.acs.org/doi/10.1021/acssuschemeng.5c00374 (accessed 2025-09-15).

(2) Lei, H.; Chen, Z.; Zhang, J.; Yu, W. Ti_3C_2Tx MXene-Assisted Solar-Driven CO_2 Adsorption and Photothermal Regeneration over Mesoporous SiO_2. Sep. Purif. Technol. **2024**, 347, 127537. https://doi.org/10.1016/j.seppur.2024.127537.

(3) Kataoka, T.; Orita, Y.; Shimoyama, Y. Photo-Thermal CO_2 Desorption from Amine-Modified Silica / Carbon Aerogel for Direct Air Capture. Chem. Eng. J. **2024**, 482, 148710. https://doi.org/10.1016/j.cej.2024.148710.

(4) Li, C.; Cao, X.; Liu, G.; Huang, L.; Chu, M.; Cheng, R.; Wang, A.; Xu, Z. Optimizing CO_2 Adsorption/Desorption via the Coupling of Imidazole and Carbon Nanotubes Paper for Spontaneous CO_2 Uptake from Ambient Air and Solar-Driven Release. Adv. Funct. Mater. **2024**, 34 (33), 2400423. https://doi.org/10.1002/adfm.202400423.

(5) Wang, D.; Sentorun-Shalaby, C.; Ma, X.; Song, C. High-Capacity and Low-Cost Carbon-Based "Molecular Basket" Sorbent for CO_2 Capture from Flue Gas. Energy Fuels **2011**, 25 (1), 456–458. https://doi.org/10.1021/ef101364c.
